# Age-related changes in the proteome and mitochondrial metabolism of rabbit adipose-derived stromal/stem cells

**DOI:** 10.1038/s41598-025-06030-9

**Published:** 2025-06-20

**Authors:** Alicia Toto Nienguesso, Juliane-Susanne Jung, Marie Alfes, Maria Schindler, Luisa Täubert, Carla Schmidt, Anne Navarrete Santos

**Affiliations:** 1 Department of Anatomy and Cell Biology, Faculty of Medicine, Martin Luther University, Halle (Saale), Germany; 2 Interdisciplinary Research Centre HALOmem, Institute of Biochemistry and Biotechnology, Charles Tanford Protein Centre, Martin Luther University Halle-Wittenberg, Halle (Saale), Germany; 3 Present Address: Parental Product Development Science and Technology Analytical, AbbVie Deutschland GmbH & Co. KG, 67061 Ludwigshafen, Germany; 4 Present Address: Department of Chemistry – Biochemistry, Johannes Gutenberg University, Mainz, Germany

**Keywords:** Ageing, Mesenchymal stem cells, Proteomics, Respiration

## Abstract

Adipose tissue is continuously regenerated by stromal mesenchymal stem cells throughout life. This study hypothesises that early age-related changes in the proteome and metabolic properties of subcutaneous (s) and visceral (v) adipose tissue-derived stromal/stem cells (ASCs) from young and old rabbits contribute to a loss of stem cell plasticity and function. To test this, the proteome and metabolic properties of ASCs from young and old rabbits were analysed using mass spectrometry-based label-free quantification and mitochondrial respiration measurements (Seahorse Mito Cell Stress Test). Both sASCs and vASCs from old rabbits exhibited comparable clusters of differentially expressed proteins. However, age-related changes were more pronounced in sASCs, suggesting that ageing affects ASCs differently depending on anatomical origin. In particular, a cluster of mitochondrial proteins in sASCs was differentially expressed with age, correlating with a shift in metabolic profile. The increase in mitochondrial respiration indicates that ageing ASCs lose their quiescent state and plasticity, leading to accelerated proliferation and differentiation. These proteomic findings were validated by Western Blot analysis, which confirmed the differential expression of key mitochondrial proteins. These results highlight the role of cellular origin in stem cell ageing and provide insights into the mechanisms underlying age-related stem cell dysfunction.

**Supplementary Information:**

The online version contains supplementary material available at 10.1038/s41598-025-06030-9.

## Introduction

Over the last decades, an increase of lifespan globally and an overall increase of elderly people could be observed, especially in industrialised countries. Therefore, besides social-economic adaption, new interventions and preventive measures against age-related diseases are needed to maintain or even expand the healthspan in old age^1–4^. It is crucial to understand regulatory mechanisms of ageing on a cellular level in favour to solve these problems. Ageing is a process that is described as a general loss of fitness and functional decline^5,6^. There are multiple interacting risk factors like stem cell exhaustion^7^, loss of proteostasis^8–11^ and mitochondrial dysfunction^12–14^, coming into play to contribute to the ageing of an organism. An additional factor is obesity, increasingly occurring in the elderly, which is also associated with age-related diseases like cardio vascular disease and metabolic syndrome^15–17^. Furthermore, advancing age causes the redistribution of fat depots in the body^18^. The loss of subcutaneous adipose tissue normally precedes the decrease of visceral fat, which can lead to a temporal accumulation around organs^19^. This goes hand in hand with changes in the cell metabolism.

The maintenance of adipose tissue homeostasis, renewal and repair throughout the lifespan is dependent on the state and function of adult adipose tissue-derived stromal/stem cells (ASCs)^7,20–22^. However, the capacity for self-renewal of these multipotent stem cell is limited and susceptible to regulatory alterations due to ageing. In general, the depletion of stem cells represents a significant contributing factor to the functional deterioration observed in organs with age^5,6^. These age-related changes in stem cells are associated with a number of factors, including metabolic and epigenetic alterations, as well as mitochondrial dysfunction^5,6,23,24^.

In order to maintain or even expand the healthspan in general and the need for interventions and/or the implication of preventive measures for age-related diseases e.g. through stem cell therapy, it is crucial to gain insight into regulatory mechanisms of stem cell ageing^25–28^. Even thought, mesenchymal stem cells (MSCs) are already investigated extensively in an ageing context, little is known about MSC-specific ageing mechanisms^29,30^. ASCs are especially of interested due to the accessibility, number and their potential to be used for regenerative interventions and measures^31–35^. In a previous study ASCs from young and (reproductive) older rabbits were investigated for age-related changes in stem cell physiology. Old ASCs showed an altered stem cell plasticity and a decrease (loss) in their adipogenic differentiation capacity^36^. Ageing negatively impacts the function of adipose tissue-derived stromal/stem cells (ASCs), which are critical for tissue maintenance and repair. However, the specific mechanisms underlying age-related changes in the proteome, metabolism, and regenerative potential of ASCs are not fully understood. In this study, we aim to investigate how early age-related changes in the proteome and metabolic properties of subcutaneous (s) and visceral (v) ASCs from young and old rabbits influence their function. We hypothesise that ageing alters the proteomic and metabolic profiles of ASCs, leading to a loss of plasticity and regenerative potential. To investigate early age-related changes in adipose-derived stromal/stem cells (ASCs), we selected a rabbit model, which mirrors human ageing more closely than smaller mammals. At 108 weeks of age, female rabbits exhibit hormonal and metabolic changes similar to the premenopausal period in women, making them an ideal model for studying the early stages of stem cell ageing^37,38^. Our focus on young (16–22 weeks) and mature (108 weeks) rabbits allows us to capture initial ASC changes before more dramatic ageing-related declines. The findings of Jung et al.^36^ using the same rabbit model further support the relevance of this experimental design. This approach is crucial for advancing regenerative medicine, especially in understanding how ageing affects stem cells in middle-aged patients.

## Results

### Proteome analysis of old ASCs

The proteome of six sASC and six vASC lines from old and young rabbits was analysed. For this, cells were lysed and proteins hydrolysed using two different sample preparation approaches, namely the single-pot, solid-phase enhanced sample preparation (SP3)^39^ and sample preparation by easy extraction and digestion (SPEED)^40^. The obtained peptides were analysed using nano-flow reversed phase liquid chromatography coupled mass-spectrometry. Relative quantification of proteins was done using a label-free quantification approach. For the analysis of the ASC proteome the two data sets (SP3 and SPEED sample preparation) were used, to optimise hits. The normal range of protein abundance in human cells lies between 1 and 10^7^ copies^41–43^. The proteome analysis of rabbit embryonic stem cells showed a coverage of fewer proteins in a previous study^44^. Another proteome analysis of rabbit vocal fold tissue identified 2990 unique FASTA-ID hits, leaving 1827 unique proteins after filtering for non-zero value label free quantification (LFQ) criteria^45^.

### Assessment of proteome data from rabbit ASCs

**Fig. 1:**
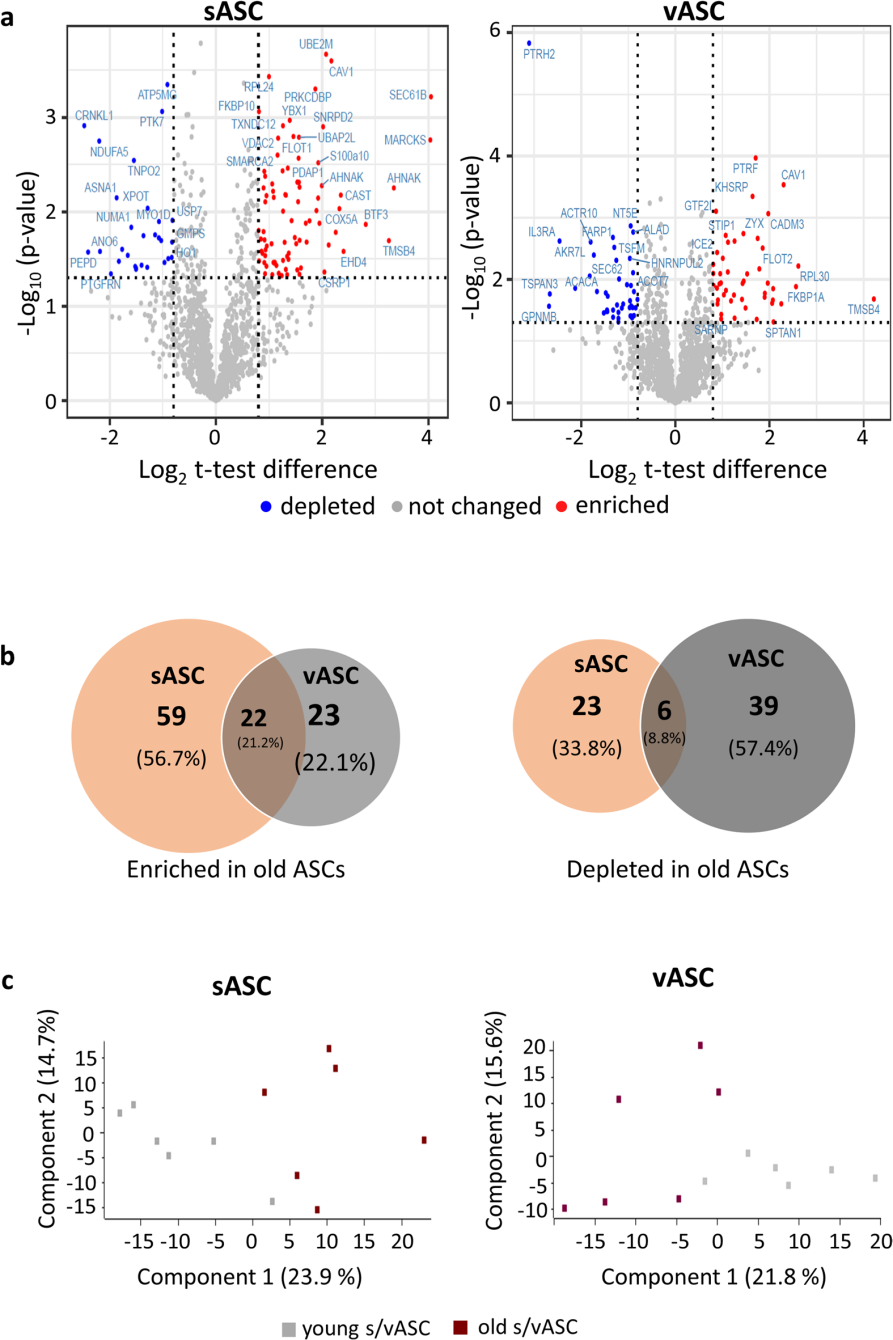
Differentially expressed proteins in old s/vASCs and principal component analysis (PCA). Six undifferentiated primary sASC and vASC cell lines from old and young rabbits were used to analyse proteome. 25 µg total protein (RIPA-lysates) of each sample were prepared using the SP3 protocol. A nano-flow reversed phase liquid chromatography coupled mass-spectrometry based label-free quantification approach was used. In (**a**) differentially expressed proteins in old s/vASCs were visualised as Volcano plots. For relative global protein abundance quantification of the different ASC cell lines, proteins with a log_2_ (fold-change) difference minimum of ± 0.8 and a *p* value ≤ 0.05 were considered as significantly up- or downregulated. (**b**) Venn diagrams were used to illustrate similarities and differences in the proteome of old sASCs and VASCs. (**c**) The PCA plots were generated using the software PERSEUS (Version).

2806 proteins could be identified (FASTA identifiers) in sASCs and 2819 vASCs respectively using the rabbit database (UniProt). For a more robust testing of differentially expressed proteins in old sASCs and vASCs, the data set was further reduced by filtering for at least four non-zero LFQ values in one group. With the employed filtering conditions, 1755 and 1832 proteins were suitable for relative quantification in subcutaneous and visceral ASCs respectively (Fig. 1a). As this study focused on the proteome of specific ASCs only, the coverage quality for these data sets was considered to be very high. The principal component analysis (PCA) shows that young and old ASCs cluster separately from each other for both sASCs and vASCs (Fig. 1c). Showing distinct differences in the proteome of ASCs during early ageing and young ASCs.

### Alterations of protein expression varies in early ageing between sASCs and vASCs

Furthermore, a test was used to identify differentially expressed proteins in old sASCs and vASCs. Proteins with a log_2_ (fold-change) minimum of ± 0.8 and a *p* value of *p* ≤ 0.05 were considered to be differentially expressed. Applying these settings 110 and 90 proteins were identified in old sASCs and vASCs, respectively (Table supplements). 21.2% of the upregulated proteins were found in old sASCs as well as old vASCs. In comparison, 8.8% of the downregulated ones were found in both (Fig. 1a,b). Thus, showing a difference in the regulation of protein expression due to the origin of the ASCs.

### STRING analysis of differentially expressed proteins in old ASCs

**Fig. 2:**
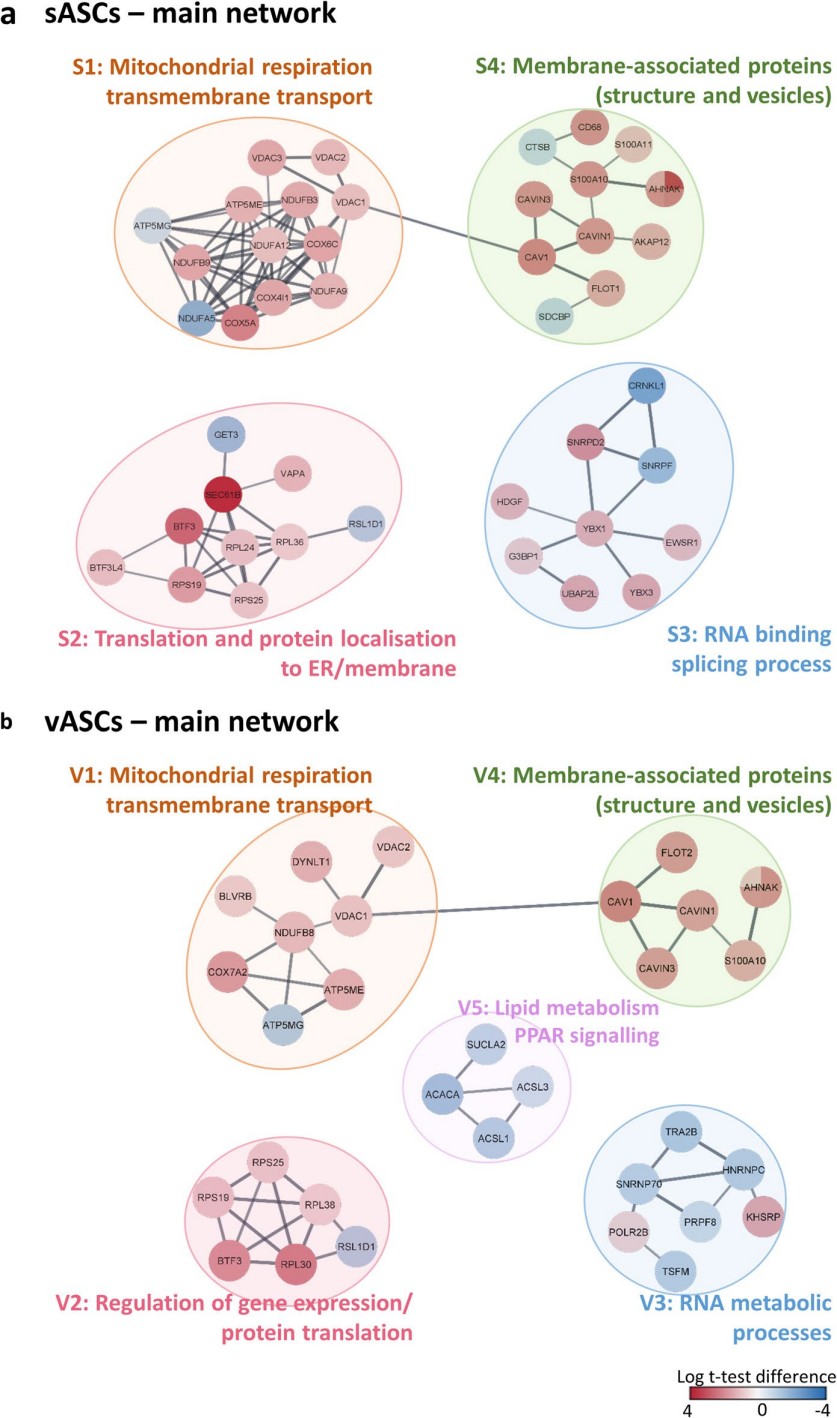
STRING analysis of differentially expressed proteins in old subcutaneous and visceral ASCs. The differentially expressed proteins (log_2_ fold-change minimum ± 0.8, *p* value ≤ 0.05) in old s/vASCs were subjected to a STRING analysis using the software Cytoscape (Version 3.9.1). A confidence cut-off of 0.7 was employed. (**a**) and (**b**) show the connected main networks. The lines between the proteins (nodes) indicate functional and/or physical interactions. The colour of the nodes, red or blue show the up or down regulation of the differentially expressed proteins. The coloured circles indicate the clusters found in old sASCs (S1–4) and vASCs (V1–5) respectively. Node colour: red upregulation, blue down regulation.

**Fig. 3:**
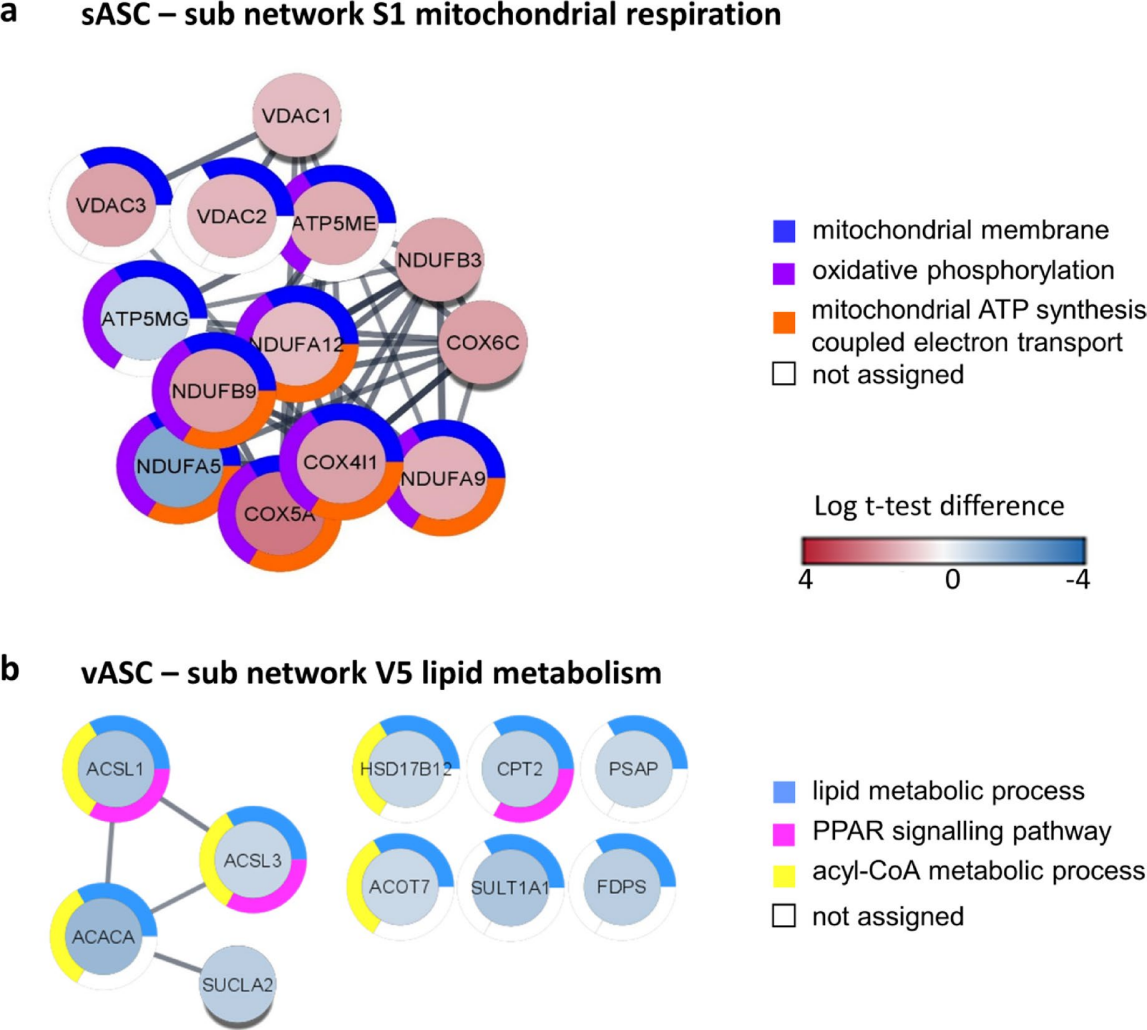
Functional STRING enrichment analysis of sub networks S1 and V5 in old ASCs. The application STRING Enrichment (Cytoscape) was used to retrieve functional information on the sub networks in old ASCs. Altered pathways were visualised using donut diagrams with data sourced from a variety of databases. The diagram (**a**) depicts the sub network mitochondrial respiration and transmembrane transport in sASCs with information sourced from gene ontology terms related to biological process, molecular function and cellular compartment. The second diagram (**b**) the sub network lipid metabolism and PPAR signalling in vASCs with data from the gene ontology terms biological process, molecular function, cellular compartment and KEGG pathways. Node colour: red upregulation, blue down regulation.

The searching tool for the retrieval of interacting genes/proteins (STRING) is an application for network analysis and visualisation of proteomics data^46^. A STRING analysis was performed for this data set searching for clusters of regulated proteins in old ASCs compared to the young ASCs that could be matched to specific pathways (Figs. 2 and 3). The STRING Enrichment application (Cytoscape V. 3.9.1) was employed using the human database as reference due to lacking information of the rabbit database. Figure 2 shows the mostly connected main networks of clusters. These proteins were summarised in Tables 1 and 2.

**Table 1:** Differentially expressed proteins in subcutaneous ASCs.

Gene name	Main uniprot accession rabbit/human	Protein name	Old sASCs versus young sASCs			
			Fold Change	Log2 t-test diff	*p* value	− Log10 *p* value
Mitochondrial respiration associated proteins (mitochondrial proteins) (cluster S1)						
ATP5ME	G1U6B4	ATP synthase subunit e, mitochondrial	2.53	1.34	0.030	1.53
ATP5MG	U3KM71	ATP synthase subunit g, mitochondrial	0.53	− 0.91	0.0004	3.35
COX4I1	G1SG11;Q9TTT8	Cytochrome c oxidase subunit 4 isoform 1, mitochondrial	2.89	1.53	0.041	1.38
COX5A	G1TZN7	Cytochrome c oxidase subunit 5A, mitochondrial	5.01	2.32	0.009	2.03
COX6C	G1TPV7	Cytochrome c oxidase subunit 6C	2.97	1.57	0.046	1.34
NDUFA5	A0A5F9C8N4	NADH dehydrogenase [ubiquinone] 1 alpha subcomplex subunit 5	0.22	− 2.20	0.002	2.75
NDUFA9	G1TM60	NADH dehydrogenase [ubiquinone] 1 alpha subcomplex subunit 9	2.40	1.26	0.033	1.49
NDUFA12	A0A5F9CX27;A0A5F9DTM7	NADH dehydrogenase [ubiquinone] 1 alpha subcomplex subunit 12	2.09	1.06	0.046	1.33
NDUFB3	G1T0J4	NADH dehydrogenase [ubiquinone] 1 beta subcomplex subunit 3	2.80	1.48	0.019	1.73
NDUFB9	G1TQG1;A0A5F9D950	NADH dehydrogenase [ubiquinone] 1 beta subcomplex subunit 9	2.98	1.57	0.020	1.70
VDAC1	Q9TT15;A0A5F9D2Q6	Voltage-dependent anion-selective channel protein 1	2.11	1.08	0.006	2.22
VDAC2	P68003;G1SWI3	Voltage-dependent anion-selective channel protein 2	2.25	1.17	0.002	2.78
VDAC3	Q9TT13;A0A5S8HX71	Voltage-dependent anion-selective channel protein 3	2.89	1.53	0.005	2.32
Protein targeting to ER; translation (clusterS2)						
BTF3	G1TKK2;U3KMX6	Transcription factor BTF3 (Nascent polypeptide-associated complex subunit beta)	7.06	2.82	0.014	1.87
BTF3L4	G1TFI4	Transcription factor BTF3 homolog 4	2.07	1.05	0.022	1.66
GET3	A0A5F9D3H1;G1SUU2	ATPase GET3	0.27	− 1.87	0.007	2.15
RPL24	G1TH55;G1SE28	60S ribosomal protein L24	2.00	1.00	0.0004	3.43
RPL36	G1TBH2;G1TTQ5	60S ribosomal protein L36	1.75	0.80	0.016	1.80
RPS19	G1TN62	40S ribosomal protein S19	3.26	1.71	0.021	1.68
RPS25	G1TDB3;A0A5F9CQN8	40S ribosomal protein S25	1.93	0.95	0.007	2.17
RSL1D1	G1SMG0	Ribosomal L1 domain-containing protein 1	0.38	− 1.40	0.037	1.43
SEC61β	A0A5F9C445;A0A5F9D860	Protein transport protein Sec61 subunit beta	16.55	4.05	0.0006	3.22
VAPA	A0A5F9CLZ4;G1SVI9	Vesicle-associated membrane protein-associated protein A	2.39	1.25	0.0037	2.43
RNA processing (splicing processes) (cluster S3)						
CRNKL1	G1T9K0	Crooked neck-like protein 1	0.18	− 2.48	0.001	2.91
EWSR1	G1SLT2;G1TSI6	RNA-binding protein EWS	2.27	1.18	0.045	1.35
G3BP1	G1U118;G1TEW3	Ras GTPase-activating protein-binding protein 1	1.86	0.90	0.006	2.25
SNRPD2	G1TI40	Small nuclear ribonucleoprotein Sm D2	4.05	2.02	0.001	2.90
SNRPF	G1T096	Small nuclear ribonucleoprotein F	0.29	− 1.77	0.025	1.60
HDGF	G1SZR8;A0A5F9CXA1	Hepatoma-derived growth factor	2.50	1.32	0.041	1.39
UBAP2L	A0A5F9D7U8;G1TP25	Ubiquitin-associated protein 2-like	2.95	1.56	0.002	2.79
YBX1	Q28618;G1TSH8;G1U1V6	Y-box-binding protein 1	2.62	1.39	0.001	2.97
YBX3	A0A5F9D6S8;G1TS97;A0A5F9CKP3	Y-box-binding protein 3	2.95	1.56	0.005	2.31
Membrane-associated, -structuring, vesicle-associated proteins (cluster S4)						
AHNAK*	A0A5F9CJ13	Neuroblast differentiation-associated protein AHNAK (Desmoyokin)	10.19	3.35	0.006	2.25
AHNAK*	A0A5F9CTM9	Neuroblast differentiation-associated protein AHNAK (Desmoyokin)	4.77	2.25	0.016	1.78
AHNAK*	G1U7K4	Neuroblast differentiation-associated protein AHNAK (Desmoyokin)	3.98	1.99	0.005	2.28
AHNAK*	A0A5F9DNU2	Neuroblast differentiation-associated protein AHNAK (Desmoyokin)	2.85	1.51	0.022	1.66
AKAP12	G1SI22;A0A5F9D6Z9	A-kinase anchor protein 12	2.59	1.37	0.027	1.56
CAV1	Q09YN6	Caveolin-1	4.51	2.17	0.0003	3.60
CAVIN1	G1U315	Caveolae-associated protein 1 (Cavin-1) (Polymerase I and transcript release factor)	3.71	1.89	0.010	2.01
CAVIN3	G1STU4;A0A5F9C6A9	Caveolae-associated protein 3 (Cavin-3) (Protein kinase C delta-binding protein)	3.66	1.87	0.000	3.30
CD68	G1SZ64	Macrosialin (CD antigen CD68)	3.86	1.88	0.013	1.95
CTSB	A0A5F9C4V2;G1TBY1	Cathepsin B	0.51	− 0.97	0.034	1.46
FLOT1	G1TDF6;A0A5F9CP68	Flotillin-1	2.75	1.46	0.002	2.80
S100A10	Q6SQH4	Protein S100-A10	3.80	1.93	0.003	2.52
S100A11	G1SNE8	Protein S100-A11	2.08	1.06	0.005	2.30
SDCBP	G1TB50	Syntenin-1	0.47	− 1.08	0.019	1.72

List of selected differentially expressed proteins from the main STRING clusters. Proteins not belonging to those clusters are not shown. The fold change (log_2_ t-test difference) and the *p* value (− log_10_ *p* value) were calculated for old sASCs.

*AHNAK: there are several entries in the rabbit Uniprot database. An alignment showed that there are minor changes in the protein sequences (single amino acids) or variations in the length (supplementary data 1). There is a high probability that these are the same protein or isoforms of AHNAK.

**Table 2:** Differentially expressed proteins in visceral ASCs.

Gene name	Main uniprot accession rabbit/human	Protein name	Old vASCs versus young vASCs			
			Fold change	log2 t-test diff	*p* value	− log10 *p* value
Mitochondrial respiration (mitochondrial proteins) (cluster V1)						
ATP5ME	G1U6B4	ATP synthase subunit e, mitochondrial	2.73	1.45	0.011	1.97
ATP5MG	U3KM71	ATP synthase subunit g, mitochondrial	0.36	− 1.48	0.017	1.78
COX7A2	A0A5F9DIF6	Cytochrome c oxidase subunit 7A2, mitochondrial	3.72	1.90	0.019	1.71
NDUFB8	G1SEH7	NADH dehydrogenase [ubiquinone] 1 beta sub complex subunit 8, mitochondrial	2.37	1.24	0.018	1.74
BLVRB	A0A5F9CAX1;A0A5F9CSV5	Flavin reductase (NADPH) (FR); Biliverdin Reductase B	1.84	0.88	0.014	1.86
DYNLT1	G1T4I7	Dynein light chain Tctex-type 1	2.68	1.42	0.021	1.68
VDAC1	Q9TT15;A0A5F9D2Q6	Voltage-dependent anion-selective channel protein 1	2.04	1.03	0.018	1.75
VDAC2	P68003;G1SWI3	Voltage-dependent anion-selective channel protein 2	1.96	0.97	0.011	1.94
Translation/gene expression (cluster V2)						
BTF3	G1TKK2;U3KMX6	Transcription factor BTF3 (Nascent polypeptide-associated complex subunit beta)	4.76	2.25	0.025	1.60
RPL30	A0A5F9DSQ7;G1TDL2	60S ribosomal protein L30	6.11	2.61	0.006	2.22
RPL38	G1U001;G1U4G2	60S ribosomal protein L38	1.75	0.81	0.006	2.24
RPS19	G1TN62	40S ribosomal protein S19	2.16	1.11	0.008	2.12
RPS25	G1TDB3;A0A5F9CQN8	40S ribosomal protein S25	1.91	0.94	0.01	1.93
RSL1D1	G1SMG0	Ribosomal L1 domain-containing protein 1	0.35	− 1.52	0.035	1.46
Transcription/RNA processing (cluster V3)						
HNRNPC	G1T4K6;A0A5F9CWP2	Heterogeneous nuclear ribonucleoproteins C1/C2	0.40	− 1.32	0.04	1.39
KHSRP	G1TJG3	Far upstream element-binding protein 2	3.12	1.64	0.0005	3.35
POLR2B	G1TBH1;A0A5F9DNJ1	DNA-directed RNA polymerase II subunit RPB2	1.75	0.81	0.008	2.10
PRPF8	G1SCK0	Pre-mRNA-processing-splicing factor 8	0.53	− 0.91	0.03	1.54
SNRNP70	G1T8P3;A0A5F9D626	U1 small nuclear ribonucleoprotein 70 kDa	0.43	− 1.21	0.05	1.31
TRA2B	A0A5F9DQE0;G1U9B4	Transformer-2 protein homolog beta	0.37	− 1.44	0.03	1.48
TSFM	A0A5F9DU46;G1T3G8;A0A5F9D5I7	Elongation factor Ts, mitochondrial	0.41	− 1.30	0.003	2.52
Membrane-associated, -structuring, vesicle associated (cluster V4)						
AHNAK*	A0A5F9CJ13	Neuroblast differentiation-associated protein AHNAK (Desmoyokin)	4.20	2.07	0.022	1.66
AHNAK*	A0A5F9CTM9	Neuroblast differentiation-associated protein AHNAK (Desmoyokin)	3.90	1.96	0.012	1.94
AHNAK*	G1U7K4	Neuroblast differentiation-associated protein AHNAK (Desmoyokin)	3.73	1.90	0.017	1.77
AHNAK*	A0A5F9CB35;A0A5F9DP01	Neuroblast differentiation-associated protein AHNAK (Desmoyokin)	1.93	0.95	0.008	2.09
CAV1	Q09YN6	Caveolin-1	4.92	2.30	0.0003	3.53
CAVIN1	G1U315	Caveolae-associated protein 1 (Cavin-1) (Polymerase I and transcript release factor)	3.26	1.71	0.0001	3.97
CAVIN3	G1STU4;A0A5F9C6A9	Caveolae-associated protein 3 (Cavin-3) (Protein kinase C delta-binding protein)	3.44	1.78	0.007	2.17
FLOT2	G1SP89;G1TFZ5	Flotillin-2	3.62	1.85	0.003	2.51
S100A10	Q6SQH4	Protein S100-A10	2.72	1.45	0.002	2.74
Lipid metabolism (V5)						
ACACA	A0A5F9DAT8;G1T7I3	Acetyl-CoA carboxylase 1	0.28	− 1.82	0.009	2.05
ACSL1	G1SPB6	Long-chain-fatty-acid-CoA ligase/synthetase 1	0.36	− 1.46	0.033	1.48
ACSL3	A0A5F9CLI5; G1T8H1	Long-chain-fatty-acid-CoA ligase/synthetase 3	0.52	− 0.94	0.013	1.90
SUCLA2	G1U276	Succinate–CoA ligase [ADP-forming] subunit beta, mitochondrial	0.43	− 1.21	0.043	1.37

List of selected differentially expressed proteins from the main STRING clusters. Proteins not belonging to those clusters are not shown. The fold change (log_2_ t-test difference) and the *p* value (− log_10_ *p* value) were calculated for old vASCs.

*AHNAK: there are several entries in the rabbit Uniprot database. An alignment showed that there are minor changes in the protein sequences (single amino acids) or variations in the length (Supplementary data 1). There is a high probability that these are the same protein or isoforms of AHNAK.

### Clusters of differentially expressed proteins sASCs versus vASCs

The clusters of differentially expressed proteins in old sASCs showed mostly an upregulated protein expression (Fig. 2, Table 1). The main network for old sASCs was divided in four clusters: (S1) mitochondrial respiration (mitochondrial proteins), (S2) translation and protein localisation to ER, (S3) RNA binding and splicing processes and (S4) membrane-associated (vesicle and transport associated) proteins (Figs. 2a and 3a, Table 1). The analysis of old vASCs showed less connected but similar clusters. In general, there were more downregulated proteins in old vASCs (Fig. 1b). The main network for old vASCs was divided in five clusters: (V1) mitochondrial respiration (mitochondrial proteins), (V2) translation and gene expression, (V3) transcription and RNA processing, (V4) membrane-associated (vesicle and transport associated) proteins and (V5) lipid metabolism (Figs. 2b and 3b, Table 2).

### Age-related increase in mitochondrial respiratory chain proteins of complex I and IV in old ASCs

**Fig. 4:**
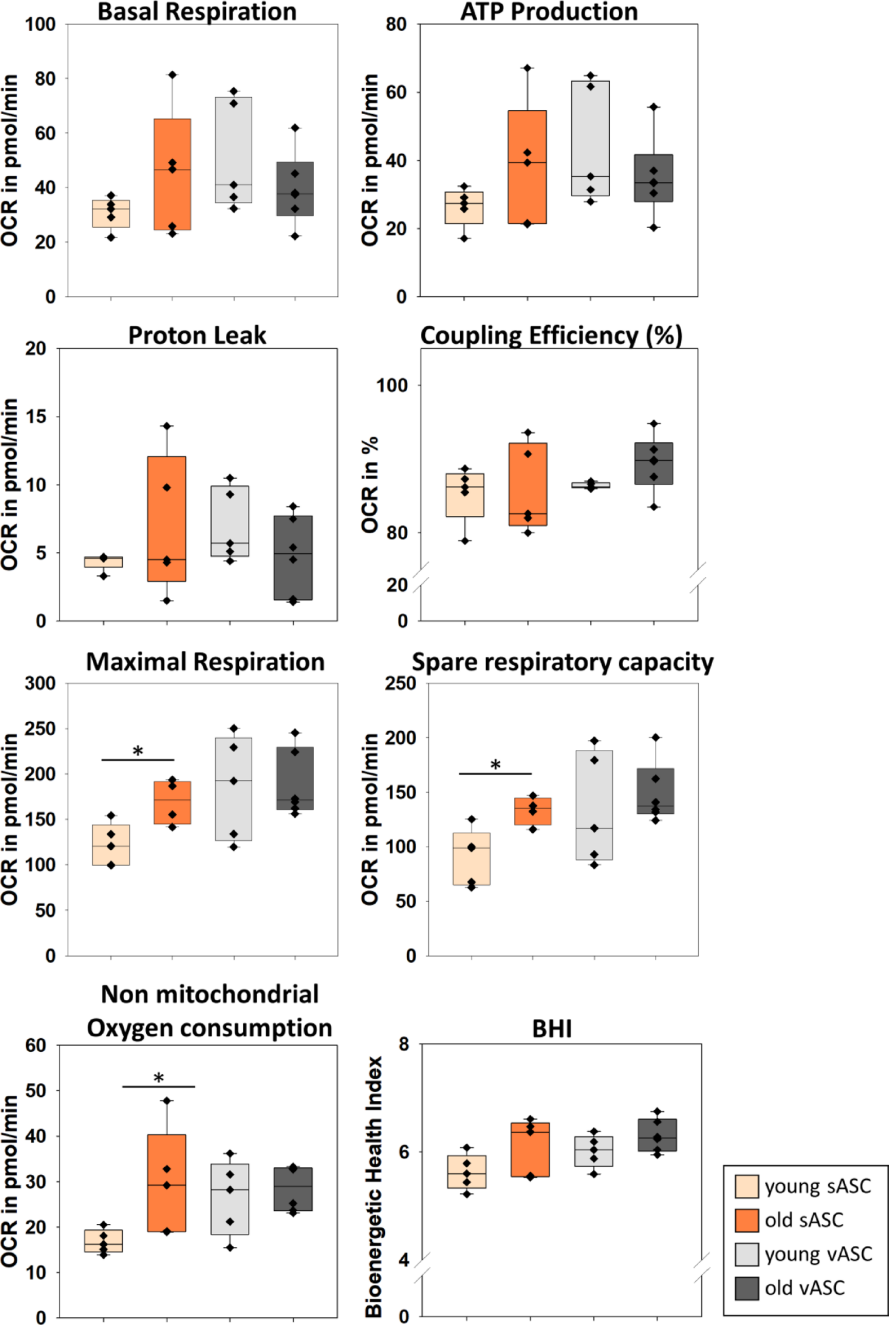
Mitochondrial respiration in undifferentiated primary ASCs. Five to six primary cell lines of each ASC group of subcutaneous and visceral adipose tissue were analysed using the Seahorse XF Mito Cell Stress Test (Agilent). The cells were used to measure the oxygen consumption rate (OCR) for the baseline and subsequently after the injection of oligomycin, FCCP and rotenone/antimycin A/Hoechst33342. Additional parameters were calculated using the Wave software. Student’s t-tests were performed *p ≤ 0.05, n = 4–6 N = 3. s/vASC subcutaneous/visceral ASC

**Fig. 5:**
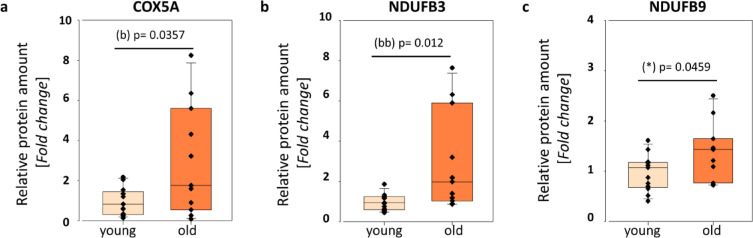
Validation of proteomics data for individual proteins COX5A (**a**), NDUFB3 (**b**) and NDUFB9 (**c**) by Western blotting in old sASCs. The box plots display fold changes of the indicated protein amounts in comparison to young sASCs. Western blotting was performed as described in the Methods section, and protein levels were quantified by comparing the adjusted intensities of protein-specific bands detected by antibodies (SFig. 3) and normalised to the total protein intensities, as assessed by Ponceau S staining (SFigs. 4 and 5). Western blots were performed on 24 samples, with n = 13 for young and n = 11 for old sASCs. Statistical significance is indicated as follows: (*): *p* ≤ 0.05 (Student’s t-test); (b): *p* ≤ 0.05 (Welch’s t-test); (bb): *p* ≤ 0.05 (Mann–Whitney Rank Sum test).

There was a major cluster observed with differentially expressed mitochondrial proteins. The mitochondrial cluster (S1) in old sASCs contains supernumerary subunits from respiratory chain complex I and IV (COX4l1, COX5A, COX6C, NDUFA5, NDUFA9, NDUFA12, NDUFB3 and NDUFB9), the ATP synthase (ATP5ME, ATP5MG) and voltage dependent anion carrier (VDAC1–3). All proteins except APT5MG and NDUFA5 were upregulated. The enrichment of proteins of the respiratory chain is supported by the metabolic analysis of sASCs (Figs. 3a and 4, Table 1). Additionally, western blot analysis was used to independently investigate, whether the proteome data could be verified (Fig. 5). The mitochondrial respiration cluster (V1) in old vASCs was smaller also containing subunits of complex I and IV of the respiratory chain and the ATP synthase (COX7A2, NDUFB8, ATP5ME and ATP5MG), the voltage dependent anion carrier VDAC1–2 and the proteins BLVRB and DYNLT1. The expression of the ATP synthase subunits was regulated as in the old sASCs (Fig. 2b, Table 2).

### Upregulation of factors impacting gene expression and the SEC61-translocon

Cluster S2 contained ribosomal proteins (RPL24 & 36, RPS19 & 25, and RSL1D1) and the general transcription factor BTF3 and its paralog BTF3L4. Furthermore, it also included the highly upregulated SEC61β which is a part of the SEC61-translocon as well as the ATPase GET3 and the VAMP-associated protein A (VAPA). The proteins in S2 were upregulated except for RSL1D1 and GET3. There was also an upregulation of ribosomal proteins in old vASCs which were included in cluster V2 (RPL30 & 38 and RPS19 & 25, RSL1D1) as well as the general transcription factor BTF3.

### RNA processing is affected in old sASCs and vASCs

Cluster S3 generally included proteins involved in RNA binding and processing. They belong to different spliceosome complexes and are involved in transcription (CRNKL1, EWSR1, G3BP1, HDGF, SNRPD2, SNRPF, UBAP2L, YBX1 and YBX3). The proteins of cluster S3 were mostly upregulated in old sASCs with the exception of CRNKL1 and SNRPF. The proteins YBX1 and XBX3 sparked particular interest as they are known to be involved in regulating specific respiratory chain complex subunits. In old vASCs (cluster V3) differentially expressed proteins associated with transcription and RNA processing (splicing) were down regulated (HNRNPC, KHSRP, POLR2B, PRPF8, SNRNP70 and TRA2B) except the RNA polymerase II subunit B (POLR2B) and the KH type- splicing regulatory protein (KHSRP).

### Age-related increase of AHNAK1 and caveola associated proteins in old ASC

Cluster S4 and V4 were similar, containing loosely associated proteins (Fig. 2a,b and Tables 1 and 2). The majority were membrane-associated and involved in lipid rafts (caveola), focal adhesion, cytoskeletal dynamics and signal transduction. AHNAK1 was highly upregulated in both old sASCs and vASCs, which could impact the downstream fate during differentiation of the ASCs, as AHNAK 1 is known to play a role in adipogenic differentiation. Furthermore, caveola associated protein (CAV1, CAVIN1, CAVIN3, FLOT1, FLOT2) were increased which can also be involved in age-related changes. Cluster V4 was similar to cluster S4 containing only fewer proteins with similar functions.

### Lipid metabolism-related proteins decreased in old vASCs

Cluster V5—a small but distinct cluster of down regulated proteins related to lipid metabolism and PPAR signalling (ACSL1, ACSL3, and ACACA and SUCLA2) could be observed in old vASCs (Fig. 3b, Table 2).

### Metabolic analysis: old sASCs show changes in mitochondrial respiration

To further analyse, whether the age of the stem cell donor has an impact on the overall metabolic profile of ASCs, metabolic parameter needed to be investigated. The mitochondrial respiration has been shown to have a major impact on the differentiation and proliferation of human MSCs^47^. Another study investigated the bioenergetic potential of ASCs from different anatomical locations and found that there were no apparent origin-dependent differences^48^. To investigate potential age-dependent changes in the mitochondrial respiration of young compared to old ASCs a Seahorse XF Mito Cell Stress Test (Agilent) was performed. Five to six primary cell lines of each ASC group, isolated from visceral and subcutaneous adipose tissue, were analysed. The oxygen consumption rate (OCR) was measured in triplets for the baseline and subsequently after the injection of oligomycin, carbonyl cyanide-*p*-trifluoromethoxyphenylhydrazone (FCCP) and rotenone/antimycin A/Hoechst33342, which target different complexes of the mitochondrial respiratory chain. Using these OCR values, further parameters were calculated as described by the manufacture (Supplement data 2).

### Increase of mitochondrial respiratory activity in sASCs in early ageing

The basal mitochondrial respiration, ATP production, proton leak and coupling efficiency (%) appeared to be quite similar between young and old ASCs. However, the median of old sASCs for basal respiration (Fig. 4*p* = 0.217) and ATP production (Fig. 4*p* = 0.213) show a tendency for an increase of these parameters compared to young sASCs. This changes maybe be more apparent and statistically significantly increased at a later stage in ASCs from geriatric animals. The main observation of the metabolic data from the assay showed a significant increase for maximal respiration (*p* = 0.0209), spare respiratory capacity (*p* = 0.0210) and non-mitochondrial oxygen consumption (*p* = 0.0467) in old sASCs compared to the young control group (Fig. 4). In contrast, old ASCs from visceral origin showed no significant changes of all parameters compared to the young group (Fig. 4). Regarding the different adipose tissue origins, we observe a tendency of slightly elevated OCR of young and old vASCs for maximal respiration, spare respiratory capacity and non-mitochondrial oxygen consumption compared to young sASCs (Fig. 4) which is not significant so far. Additionally, the Bioenergetic Health Index (BHI) was calculated, to further evaluate the condition of the old ASCs compared to the young controls. At this stage no changes were observed between the groups, yet (Fig. 4).

### Validation differentially expressed mitochondrial proteins COX5A, NDUFB3, and NDUFB9 by immunoblotting

In the process of evaluating the experimental proteomics data a western blot analysis was performed (Fig. 5; SFigs. 3 to 5). As we were interested in the observed age-dependent changes of the respiration representative proteins focusing on differentially expressed proteins of cluster S1, namely COX5A, NDUFB3 and NDUFB9 were chosen. 13 and 11 individual young and old sASC lines were employed to obtain protein samples. 25 µg protein (RIPA-lysates) of each respective sample were used to validate our findings. A statistically significant increase in the fold change was observed for the three target proteins, COX5A (*p* = 0.0357), NDUFB3 (*p* = 0.012) and NDUFB9 (*p* = 0.0459) (Fig. 5) from the cluster S1. Hence, we were able to independently verify the proteome data. This was further supported by our physiological data of the increase of mitochondrial respiratory parameters in old sASCs.

## Discussion

The present study was designed to analyse changes in the proteome during early ageing of adult undifferentiated ASCs, as well as associated metabolic alterations, with the objective of characterising specific stem cell ageing processes during maturation.

One part was the proteome analysis of young and old ASCs (during maturation). The proteome coverage achieved in this study was considered robust, based on the available rabbit proteome data^44,45^. Based on the STRING analysis several pathways were identified that may have an impact on the quiescent state of adult stem cells and on their function and physiology in the context of ageing. The subsequent discussion will focus on differentially expressed mitochondrial proteins and their influence on stem cell function and physiology. So far, several studies showed the impact of mitochondrial dysfunction on stem cell ageing, which is not only specific to ASCs but observed over a variety of mesenchymal stromal/stem cells (MSC), such as bone marrow-MSCs^49–51^. MSC are primarily glycolytic cells, generally suppressing the oxidative phosphorylation to maintain a quiescent state^52,53^. This is crucial for self-renewal in adult stem cells. The increase of mitochondrial respiration in MSCs leads to the propagation of proliferation and differentiation^54^. A recent study showed an increase of mitochondrial respiration from precursor cells, preadipocytes, to fully differentiated adipocytes, which is contrary to maintain the quiescent state. Additionally, it has been shown that modulating mitochondrial respiration can help mitigate age-related metabolic dysfunction^47^. In old sASCs several mitochondrial proteins were altered. The cluster contains supernumerary subunits from respiratory chain complexes and the ATP synthase. The proteins were enriched except for NDUFA5 and ATP5L (Figs. 2a and 3a, Table 1). Furthermore, the catalytic subunit MT-CO2 also showed an increase by almost doubling (logFC 0.95, *p* = 0.052). The upregulation of this cluster correlates with the metabolic analysis showing a pronounced upregulation of maximal respiration and spare respiratory capacity in sASCs from old donors (Fig. 4). These changes indicate the loss of quiescence and stem cell functionality in old sASCs. In contrast, the changes in mitochondrial protein expression were less pronounced in old vASC (Fig. 2b, Table 2). This aligns with the metabolic data, where no significant changes were observed in old vASCs (Fig. 4, Table 2). Although, the median for the maximal respiration was slightly decreased in old vASCs compared to young controls, which may be an indication for an increase in stress susceptibility^55^.

Furthermore, two particular proteins have been demonstrated to influence adipogenic differentiation and mitochondrial function, making them important factors in ASC ageing. AHNAK1 (neuroblast differentiation-associated protein), upregulated in old s/vASCs (Tables 1 and 2), is essential for BMP4/SMAD-mediated adipogenesis. It plays a role in mitochondrial regulation and age-related changes in mitochondrial morphology and function^56^, as demonstrated in Ahnak1 knockout mice by testing the differentiation capacity of primary ASC and C3H10T1/2 control cells transfected with siRNA. Moreover, the expression of adipogenic markers including PPARγ, C/EBPβ and aP2 were markedly reduced^57^. In addition, further studies observed a link of AHNAK1 gene expression and ageing^58,59^. In cardiomyocytes from Ahnak1 knock-out mice AHNAK1 was found to play a role in the age-related regulation of mitochondrial morphology, function and activity. These functions have been corroborated in human AC16 cells^60^. The regulatory mechanisms of AHNAK1 in the ageing of undifferentiated ASCs remain unclear and warrant further investigated, particularly regarding mitochondrial function in aged ASCs.

Calpastatin (CAST) was highly upregulated in old sASCs. It is an endogenous inhibitor of calpain (CAPN1–15). These proteins are central to a calcium-dependent proteolytic system^61^. Calpastatin as well as calpain and the matrix metalloproteinase 15 (MMP-15) influence the fate of human ASCs (invasion) in tumorigenesis in the context of obesity^62^. In addition, the CAST expression has also been shown to influence the mitochondrial morphology^63^. For our study the upregulation could indicate a potential role in regulating mitochondrial morphology and function in aged sASCs. The proteome analysis gave rise to more putative factors for age-dependent regulatory mechanisms influencing the mitochondrial respiration. The Y-box binding proteins 1 and 3 (YBX1, YBX3; cluster S3) were upregulated in old sASCs and are known to be translational regulators of specific mRNAs of the oxidative phosphorylation like COX5A and NDUFA9, acting as a translational suppressor^64^. The observed upregulation of YBX1 and YBX3, which bind similar mRNAs, may play a regulatory role in mitochondrial respiration subsequently impact stem cell quiescence.

The upregulation of mitochondrial respiration and non-mitochondrial oxygen consumption in old sASCs (Fig. 4) is associated with increased reactive oxygen species (ROS) levels, indicating cellular stress and contributing to a decline in stem cell health and bioenergetic function^55^. As we did not observe any changes in die BHI (Fig. 4), it may lead to a decline of the BHI in more aged ASCs. Additionally, there was an up regulation of VDAC1, VDAC2 and VDAC3 in old ASCs (Fig. 2 Tables 1 and 2) which play a role in ROS regulation, mitochondrial function regulation and essential cell processes as recent studies showed^65,66^. More specific, these processes include regulatory mechanisms for ATP production^67^, OMM dependent CA^2+^ flux, to maintain balance in ROS impairment, autophagy and apoptosis^68^. The alteration in the VDAC expression could also be an age-related regulatory mechanism that impacts the changes observed in the mitochondrial respiration.

There were further indications that the lipid metabolism and caveolae were affected during early ageing as specific clusters of proteins were differently expressed. In old vASCs, the expression of fatty acid metabolism proteins (ACSL1, ACSL3, ACACA, and SUCLA2) was decreased, affecting the esterification of long-chain fatty acids to acyl-CoA derivatives, which are critical for mitochondrial β-oxidation^69^. Disturbances in the β-oxidation of long-chain fatty acids could affect downstream processes such as glycolysis and may cause lipid-induced stress in mitochondria and the cells^70,71^. Moreover, ACSL1 and ACSL3 have the potential to influence the lipid metabolism through the PPARγ pathway^72,73^. A consensus that changes in the lipid metabolism play a role in ageing of MSCs is apparent across a number of publications in recent years^6,74^. In addition, SUCLA2 is the ATP-generating subunit of the succinyl-CoA synthase, which is part of the mitochondrial tricarboxylic acid cycle. It was observed that SUCLA2 plays a role in the development of mitochondrial disorders^75^. Changes in the expression levels may additionally impact the ageing processes of visceral ASCs.

Both old visceral and subcutaneous ASCs showed an upregulation of caveolae-associated proteins (clusters S4 and V4). Particularly, the levels of CAV-1 (CAV-protein) and CAVIN-1 were significantly elevated which are found in caveolae, a distinctive type of membrane microdomains. The CAV-proteins play a significant role in processes such as membrane restructuring, signal transduction and endocytosis^76,77^. A number of studies have identified a correlation between CAV-1 upregulation and the promotion of cellular senescence which is a key factor in the ageing process^78,79^. An increase of CAVIN-1 expression in human murine fibroblasts has been linked to elevated oxidative stress and stress-induced premature senescence, which occurs due to the interaction with CAV-1^80^. CAVIN-3 was upregulated as well (Fig. 2, Tables 1 and 2) in old ASCs. It is known that the increase of CAVIN-3 exerts an influence on the adipocyte differentiation promoting these processes and modulating the expression of adipogenesis-related genes. This includes peroxisome proliferator- activated receptor gamma (PPARγ), FASW, adipocyte protein 2 (aP2), adiponectin (AdipoQ) and preadipocyte factor 1 (Pref-1)^81^. These findings indicate that caveolae may be involved in age-related regulation and promotion of senescence and downstream differentiation processes in old ASCs.

The elevation of vesicle and transport-associated proteins in old sASCs and vASCs may be a result of the intra- and extracellular changes such as the increase of translation, requiring a higher volume of vesicle-dependent and independent targeted transport processes. It has been demonstrated that alterations in extracellular vesicles influence the ageing cellular milieu and are associated with age-related pathologies^82^.

Maintaining proteostasis and thus managing translation is a factor which plays a role in ageing. Several studies have shown that translational processes both are affected and can influence ageing. The inhibition of global protein synthesis is generally associated with an increase in lifespan^6,83,84^. The proteome data showed a general upregulation of proteins involved in translational processes in old ASCs (11 proteins, clusters S2 and S3; 5 proteins, clusters V2 and V3). Therefore, the observed increase could have an overall negative impact on old sASCs and vASCs, accelerating ageing processes. Additionally, there was an impact on co-translational translocation of proteins to the ER in old sASCs, as evidenced by the pronounced upregulation of SEC61β, a subunit of the SEC61-translocon (Fig. 2, Table 1). The SEC61–translocon plays a pivotal role in maintaining ER homeostasis and in ER stress regulation^85^. This upregulation could also be in part due to the observed overall increase of translation in old sASCs and may provide further insight into mechanisms of ageing in stem cells as ER stress has also been linked to ageing and longevity^6,86^. So far, the study could show that the observed age-related changes in protein expression are not isolated but correlate with physiological alterations in the adult stem cells. This illustrates again, that ageing is the sum of several factors as published by Lopéz-Otín et al.^5,13^. These alterations may indicate that early ageing leads to ASCs shifting to a preactivated state similar to precursor cells, which would lead to stem cell exhaustion over time.

Considering the current study and previous findings, it appears that there are differences in age-related regulations and mechanisms between subcutaneous ASCs and visceral ASCs.

This point is of vivid discussion, as many contrary findings can be found in the literature. Some stating that there is no significant impact of ageing or patient age on the ASCs^87^, but rather between different mesenchymal stems cell types (MSC) (bone marrow-, muscle and adipose-derived)^88,89^. This implicates that the type of the MSC is of higher importance compared to anatomical origin. Whereas a different study showed that age and anatomical region impact the function in subcutaneous ASCs^90^. Additionally, it has been demonstrated that the stem cell plasticity of old ASCs was affected by early ageing and their adipogenic differentiation capacity was reduced^36^. Furthermore, this study showed the general alteration of ageing marker at the transcriptional level in old sASCs. A significant decrease of APOE, FGF2 and Sirt1 mRNA amounts. In addition, the protein amounts of specific age marker APOE (metabolic longevity sensor), ATG7 (autophagy marker) and PTEN (cell cycle and self-renewal) were significantly increase in old vASCs^36,91–93^. This could indicate tissue specific rescue mechanisms in old ASCs to counter age-related alterations and maintain the stem cell character.

Although there is evidence of differing effects and mechanisms of ageing in ASC depending on the anatomical location, these analyses were performed in ASCs from the rabbit. Given the considerable similarities and conservation of processes such as fatty acid metabolism between humans and rabbits, primary rabbit ASCs represent an established and valuable model for investigating these processes and their implications in ageing.

## Conclusion

The process of ageing is characterised by the accumulation of regulatory and functional deterioration over time. This study focused on early age-related changes in ASCs from 2 to 3-year-old rabbits, which corresponds to a human age of 35–45 years. Although, it is not a geriatric group (advanced age < 65 years), we demonstrated that age-related changes can already be observed at this stage of age. The differentially expressed proteins and the associated signalling pathways highlight the role of mitochondrial metabolism and dysfunction in the ageing of adult stem cells. The metabolic profile of ASCs derived from subcutaneous adipose tissue exhibited more pronounced age-related alterations. In contrast, visceral ASCs showed less pronounced changes, indicating that these cells may possess stronger regulatory mechanisms or protections, potentially related to intracellular pathways. These findings suggest that the anatomical origin of adipose tissue may play a critical role in determining the extent of early age-related changes in stem cells.

## Methods

### Cell culture of rabbit ASCs

Cell experiments were performed in established in-vitro primary ASC lines (source of cells: New Zealand White, ZIKA hybrid, female, gravid day 6 *post coitum)* described in the paper by Jung et al.^36^. The origin of the used primary ASCs was from both visceral (perirenal adipose capsule) and subcutaneous (inguinal subcutis) adipose tissue and from young females (16–22 weeks) or from females older than 108 weeks of age (equivalent to a human age of 35–45 years). Each rabbit served as a donor for one sASC and one vASC line, respectively, as described by Jung et al.^36^. The cells were cultured in accordance with standard cell culture conditions at 37 °C with 5% CO_2_ (20% N_2_) in a cell culture medium (DMEM + 10% FBS, 1% penicillin/streptomycin, 1% amphotericin B) The passage 0 cell culture medium was additionally supplemented with 1% gentamycin. The undifferentiated cells were harvested in passages 2–5 for further investigation. Frozen ASCs were employed to secure groups of 6–7 cell lines each, with the ASCs from young donors serving as the control for the ASCs from old donors.

### Sample preparation for LC–MS/MS

#### Protein preparation with RIPA and single-pot, solid-phase-enhanced sample preparation (SP3)

For this study, protein samples from 6 to 7 separate ASC cell lines from young and old rabbits were used. Each isolated cell line was cultured separately, with no pretreatment before the proteome analysis. ASCs were disrupted in 200 μl of cold radio immunoprecipitation assay (RIPA) buffer with protease and phosphatase inhibitor (Roche, Basel, Switzerland). One sample contained 1.5 to 6 × 10^6^ cells. The protein concentration was measured using the Bradford Assay (BioRad Laboratories GmbH, Munich). Subsequently, the SP3 protocol, which is based on paramagnetic beads, was performed as previously described^39^. For this, Sera-Mag SpeedBeads (GE Healthcare, Chicago, USA) were washed three times with water. The RIPA cell lysate (25 μg total protein) was then added to the beads. Followed by reduction and alkylation of disulfide bonds with 8 mM tris(2-carboxyethyl)phosphine (TCEP) and 3 mM 2-chloroacetamide (CAA).The sample was incubated for 5 min at 95 °C. The beads with bound protein were washed three times with 80% (v/v) ethanol. For protein hydrolysis, 1 µg trypsin (Promega, Mannheim) in 25 mM ammonium bicarbonate, pH 8.5, was added at an enzyme: protein-ratio of 1:25. The sample was sonicated for 30 s in a water bath to disaggregate the beads followed by incubation at 37 °C and 1000 rpm overnight. The sample was centrifuged at 20,000×*g* for 1 min at room temperature. The supernatant containing the generated peptides was collected by separating beads and peptides using a magnet. The peptides were dried in a vacuum centrifuge.

#### Sample preparation by easy extraction and digestion (SPEED)

Furthermore, sample preparation was performed using easy extraction and digestion (SPEED). For this experiment, 6–7 cell lines from young and old rabbits each were used to collect samples for each respective experimental group (sASCs, vASCs; young and old).The second approach for cell lysis was the use of trifluoroacetic acid (TFA) as previously described^40^. The cell pellet was incubated with 4 vol. of TFA for approximately 2 min at room temperature until cells were completely lysed. For neutralisation, 10 vol. (corresponding to the amount of TFA used for cell lysis) of 2 M tris(hydroxymethyl)aminomethane were added. For reduction and alkylation of disulfide bonds 1.1 vol. (according to the amount of TFA used for cell lysis) of 29 mM tris(2-carboxyethyl)phosphine (TCEP) and 37 mM 2-chloroacetamide (CAA) mix were added and the sample was incubated for 5 min at 95 °C. The protein concentration was estimated using a DS-11 + Spectrophotometer. The cell lysate (50 µg protein) was then diluted 1:5 with water, and trypsin was added at a protein to enzyme ratio of 50:1 followed by incubation overnight at 37 °C and 600 rpm in a thermomixer. Subsequently, the peptides were desalted using Pierce Peptide Desalting Spin Columns (Thermo Scientific, Waltham, USA).

#### Peptide desalting

The peptides were desalted using Pierce™ Peptide Desalting Spin columns (Thermo Scientific, Waltham, USA) according to the manufacturer’s protocol. Briefly, the storage buffer was removed by centrifugation at 5000×*g* for 1 min. The spin column was washed twice by adding 300 µl acetonitrile (ACN) followed by centrifugation at 5000×*g* for 1 min. The column was again washed twice with 0.1% TFA (v/v) as described. The peptides were then sequentially loaded onto the spin column in 300 µl aliquots followed by centrifugation at 3000×*g* for 1 min after each loading step. After washing the column three times with 0.1% TFA (v/v) as described above, desalted peptides were eluted with 300 µl 50% (v/v) ACN and 0.1% (v/v) TFA by centrifugation at 3000×*g* for 1 min. Elution was repeated once, and the peptides were dried in a vacuum centrifuge.

### Sample analysis by LC–MS/MS

Tryptic peptides were dissolved in 2% (v/v) ACN/0.1% (v/v) formic acid (FA) and analysed by nano-flow reversed-phase liquid chromatography on a DionexUltiMate 3000 RSLCnano System (Thermo Scientific, Waltham, USA; mobile phase A, 0.1% (v/v) FA; mobile phase B, 80% (v/v) ACN/0.1% (v/v) FA coupled with a Q Exactive Plus Hybrid Quadrupole-Orbitrap mass spectrometer (Thermo Scientific, Waltham, USA). For desalting, peptides were loaded onto a trap column (μ-Precolumn C18 Acclaim™ PepMap™ 100, C18, 300 μm I.D., particle size 5 μm; Thermo Scientific, Waltham, USA) with a flow rate of 10 µl/min. The peptides were then separated with a flow rate of 300 nl/min on an analytical C18 capillary column (50 cm, HPLC column Acclaim™ PepMap™ 100, C18, 75 μm I.D., particle size 3 μm; Thermo Scientific, Waltham, USA). For proteome analysis, a gradient of 4–90% (v/v) mobile phase B over 152 min was applied. Peptides were directly eluted into the mass spectrometer.

Typical mass spectrometric conditions were: spray voltage, 2.8 kV; capillary temperature, 275 °C; data-dependent and positive ion mode. For the proteome analysis, survey full scans were acquired in the Orbitrap with a resolution of 70,000, an automatic gain control (AGC) target of 3e6, a maximum injection time of 80 ms and a scan range from 350 to 1600 m/z. Fragmentation of the 20 most intense ions with charge states of 2+ to 7+ was performed in the HCD cell employing a stepped collisional energy of 30%. MS/MS spectra were acquired with a resolution of 17,500, an AGC target of 1e5 and a maximum injection time of 150 ms. The fixed first mass was set to 105 m/z. Previously, selected ions were dynamically excluded for 30 s. The lock mass option (lock mass m/z 445.120025) was used for internal calibration in all measurements^94^. QCloud2^95^ was used to control instrument longitudinal performance.

### Proteomic data analysis and statistics

Raw data of 6–7 biological replicates of either old and young subcutaneous or visceral ASCs were searched against the rabbit UniProt database (UniProt, Proteome ID: UP000001811, 41000 entries, version date: 7th July 2021) using MaxQuant (version 1.6.17.0)^96^. Standard parameters were used: fixed modification, carbamidomethyl (cysteine); variable modifications, oxidation (methionine) and acetylation (protein N-terminus); max missed cleavage sites, 2; min peptide length, 7; max peptide mass 6000 Da; peptide FDR, 0.01; protein FDR, 0.01; enzyme, trypsin/P (cleavage C-terminal of lysine or arginine also when the C-terminal amino acid is proline). The iBAQ, MaxQuant LFQ and ‘match between runs’ options were enabled.

Further analysis was performed in Perseus (version 1.6.14.0) using the obtained ‘proteinGroups.txt’ file. First protein identifications classified as “Only identified by site”, “contaminants” and “reverse” were omitted. Then LFQ intensities of proteins were transformed to logarithmic scale with base two. Proteins with less than 5 valid values in at least one group were removed from the dataset and missing values were imputed (normal distribution, width: 0.3, down shift: 1.8). A principal component analysis was performed to gain information of the quality of the data set. A two-tailed t-test was used for identification of differentially expressed proteins. Proteins were considered to be differentially expressed when the difference was statistically significant (*p* ≤ 0.05) and the log2 (fold-change) minimum was ± 0.8

The obtained data of the t-test was visualised in a volcano plot. Volcano plots were generated in R (version 4.0.2)^97^ in RStudio (version 1.3.959)^98^ using ggrepel (version 0.9.1), ggplot2 (version 3.3.5), dplyr (version 1.0.7), svglite (version 2.0.0), openxlsx (version 4.2.4) packages.

For the differentially expressed proteins some proteins were incompletely annotated in the uniport rabbit database. Therefore, these proteins were blasted against the human uniprot database (Uniprot, Proteome ID: UP000005640, 73947 entries, version date: 2nd February 2022). The list of all differentially expressed proteins is summarised in the Supplementary data file 2.

To identify networks/pathway cluster of the data set a STRING analysis of enriched and depleted proteins in old sASCs and old vASCs was performed using Cytoscape (Version 3.9.1). To achieve a reliable analysis a confidence cut off of 0.7 was employed. For relative global protein abundance quantification of the different ASC lines, proteins with a log2 (fold-change) minimum was ± 0.8 and *p* value ≤ 0.05 according to Benjamini and Hochberg were considered as significantly up or down regulated^94^.

### Agilent seahorse XF cell mito stress test

The metabolic profile of ASCs was analysed using the Seahorse XF Mito Cell Stress Test Kit with the Seahorse XFe96 Analyzer (Agilent)^99^. The experimental set up and measurement parameters were programmed using the software Wave (Version 2.6.1). The assay was performed according to the manufacturers’ protocol.

To perform this assay, 5–6 primary ASC lines were used for each respective experimental group (sASCs, vASCs; young and old). ASCs (passage 2–5) of visceral and subcutaneous origin from young and old donors were employed. The adherent ASCs were removed from the cell culture flasks with 0.08% EDTA (37 °C). A 96 well assay plate was coated with 0.1% gelatine. 20,000 cells were used for each technical replicate. The corner wells served as blank with cell culture medium only. The cells were incubated at 37 °C, 5% CO_2_ until the Mito Cell Stress test was performed. The assay medium and chemicals, oligomycin, FCCP, rotenone/antimycin A and hoechst3342) were prepared following the manufactures’ protocol (S1 supplements). The cells were washed with 180 µl assay medium and incubated for 1 h at 37 °C without CO_2_. Oligomycin (2 µM), FCCP (1 µM) and rotenone/antimycin A/hoechst3342 (0.5 µM/20 µg/µl) were transferred in their designated ports A, B and C.

### Seahorse data analysis and statistics

The oxygen consumption rate (OCR) was recorded as triples for each measurement point for baseline, oligomycin, FCCP and rotenone/antimycin A/Hoechst3342. To normalise the OCR values the Hoechst signal for each well was used. After the assay, the following parameters were calculated: basal respiration, ATP production, maximal respiration, proton leak, spare respiratory capacity, non-mitochondrial oxygen consumption and coupling efficiency (%). In addition, the Bioenergetic Health Index (BHI) was calculated using the adjusted formula published by Chacko et al.^55^ and Maglioni et al.^100^. The data for each parameter was visualised as box plots using SigmaPlot (Version 14.5). For the statistical analysis two tailed students t-tests were performed. The parameters from old ASCs with a *p* value of *p* ≤ 0.05 were considered significantly up or down regulated. The measured OCR data is summarised in the Supplementary data file 2.

### Quantification of COX5A, NDUFB3, and NDUFB9 expression by immunoblotting

25 µg of protein from the sample RIPA lysates were used for western blot analysis. The young sASC control group had 13 samples and the old sASC group contained 11. The samples were subjected to a SDS–polyacrylamide gel electrophoresis (SDS-PAGE) run on 10–15% gradient gels and electrotransferred to nitrocellulose membranes (Cytiva, Freiburg, Germany). The membranes were stained using PonceauS (1:10) staining for 3 min, rinsed and documented for quantification normalisation of the individual samples. Membranes were blocked in Tris-buffered saline containing 0.1% (vol/vol) Triton X-100 (0.1% TBST) and 3% (wt/vol) non-fat dry milk or in bovine serum albumin (Sigma-Aldrich, Steinheim, Germany). Before blocking the nitrocellulose membranes were cut at the indicated sites (Supplementary data 1, SFig. 5). The following primary antibodies were used mouse-anti-COX5A: A-5, sc-376907, 1: 1000; mouse-anti-NDUFB3: F-12, sc-393351, 1:1000 (500); mouse-anti-NDUFB9: D-7, sc-398869, 1: 1000. The secondary antibody was used in a 1:10,000 dilution (goat-anti-mouse IgG conjugated to horseradish peroxidase, Dianova). The ChemiDoc™ Touch System and Image Lab 5.2.1 software (Bio-Rad, Hercules, CA, USA) were used for detection of specific chemiluminescence signals and subsequent quantification of the adjusted band intensities of the respective sample and target proteins. For normalisation, to correct for any loading differences between the samples, the ratio of the band intensity of the target protein to the corresponding total protein load of the sample (Ponceau S staining intensities) was used. The fold change of the relative protein amounts was calculated and visualised as boxplots for the respective target proteins. SigmaPlot was used to perform a statistic analysis (two-tailed student’s t-test) of the data, testing first for normality distribution (Shapiro–Wilk) and equal variance (Brown–Forsyth). A *p* value of *p* ≤ 0.05 was considered a statistically significant change. In case of failed normality test a Mann–Whitney-rank sum test was performed. In case of failed equal variance test a Welch’s t-test was performed.

### Sample size and statistical analysis

The sample size for each experimental group consisted of at least 5–7 individual ASC lines (with 11–13 cell lines for Western blot analysis). This sample size is sufficient to ensure adequate statistical power for detecting significant differences using a two-tailed t-test. Each group contained at least 4 data points, which is considered sufficient for a robust statistical analysis. To account for biological variability inherent to ASCs, individual cell lines exhibiting extreme outlier values were excluded following an outlier test (GraphPad). This approach ensured that only representative data as included in the final analysis.

## Electronic supplementary material

Below is the link to the electronic supplementary material.

Supplementary Material 1

Supplementary Material 2

## Data Availability

The mass spectrometry proteomics data have been deposited to the ProteomeXchange Consortium (http://proteomecentral.proteomexchange.org) via the PRIDE^101^ partner repository. The dataset identifier for the data is PXD057467 (Perseus and STRING analysis also included). The Seahorse Assay data is summarised in the supplementary data file 2.
