# Supplementary material for: Age-related changes in the proteome and mitochondrial metabolism of rabbit adipose-derived stromal/stem cells
Source: Sci Rep. 2025 Jun 20;15:20183. doi: 10.1038/s41598-025-06030-9 (PMC12181353; doi:10.1038/s41598-025-06030-9)
Supplement: Supplementary file 1 — Supplementary Material 1 [file 41598_2025_6030_MOESM1_ESM.pdf]

Age-related changes in the proteome and mitochondrial metabolism of rabbit adipose-derived stromal/stem cells

Alicia Toto Nienguesso[1], Juliane-Susanne Jung[1], Marie Alfes[2,3], Maria Schindler[1], Luisa Täubert[1], Carla Schmidt[2,4] and Anne Navarrete Santos[1*]

[1]Department of Anatomy and Cell Biology, Martin Luther University Faculty of Medicine, Halle (Saale), Germany,

[2]Interdisciplinary Research Centre HALOmem, Institute of Biochemistry and Biotechnology, Charles Tanford Protein Centre, Martin Luther University Halle-Wittenberg, Halle (Saale), Germany

[3currently:] Parental Product Development Science & Technology Analytical, AbbVie Deutschland GmbH & Co. KG, Ludwigshafen 67061, Germany

[4currently:] Department of Chemistry – Biochemistry, Johannes Gutenberg University Mainz, Germany

*correspondence: a.navarrete-santos@medizin.uni-halle.de

ORCID:

Alicia Toto Nienguesso: 0000-0002-0832-5489

Anne Navarrete Santos: 0000-0002-2176-2499

Juliane-Susanne Jung: 0000-0002-7738-4693

Carla Schmidt: 0000-0001-9410-1424

Marie Alfes: 0000-0001-5006-9573

Maria Schindler: 0000-0002-2151-767X

AHNAK1 alignment of rabbit sequences

Tool: COBALT Constraint-based multiple alignment tool(NCBI)

**Alignment Parameters**	
Gap penalties	-11,-1
End-Gap penalties	-5,-1

**CDD Parameters**	
Use RPS BLAST	on
Blast E-value	0.005
Find Conserved columns and Recompute	on

**Query Clustering Parameters**	
Use query clusters	On
Word Size	4
Max cluster distance	0.8
Alphabet	SE-B15

Aligned sequences:

Accession	Description
Query_10001	G1U7K4S_RABIT AHNAK nucleoprotein OS=Oryctolagus cuniculus OX=9986 PE=4 SV=2
Query_10002	A0A5F9CTM9_RABIT AHNAK nucleoprotein OS=Oryctolagus cuniculus OX=9986 PE=4 SV=1
Query_10003	A0A5F9CJ13_RABIT AHNAK nucleoprotein OS=Oryctolagus cuniculus OX=9986 PE=4 SV=1
Query_10004	A0A5F9DNU2_RABIT AHNAK nucleoprotein OS=Oryctolagus cuniculus OX=9986 PE=4 SV=1
Query_10005	A0A5F9DP01_RABIT AHNAK PDZ domain-containing protein OS=Oryctolagus cuniculus OX=9986 PE=4 SV=1

Visual alignment the identified AHNAK sequences:

NCBI Multiple Sequence Aligment Viewer, Version 1.25.2

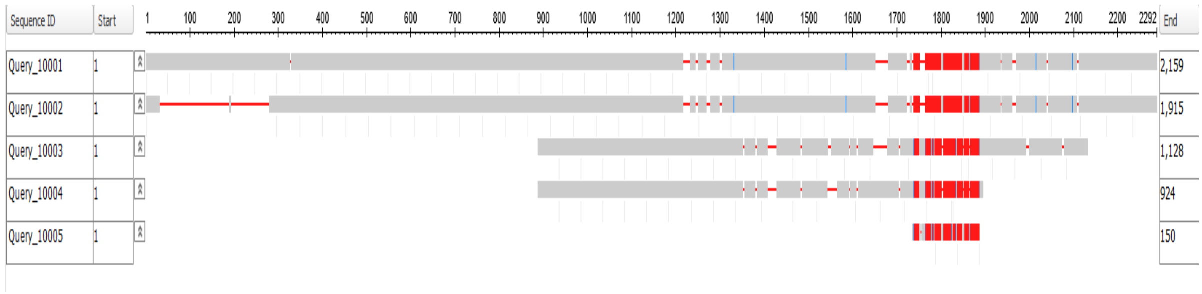

Aligned amino acid sequence of AHNAK:

Query_10001 MFSSCDVAVSPCLLSRVPFPRRLPGPLSVMVPASLGTRHAKGLDLGGKGEVQAPVSSSLGG

Query_10002 MFSSCDVAVSPCLLSRVPFPRRLPGPLSVMV-----------------------------

Query_10003 --

Query_10004 --

Query_10005 --

Query_10001 GAVEVQGPSLESGDGGKIKIPTMKVPKFAVLTGPEGQAPEAGLSVSAPEFSVGHKGGRLGS

Query_10002 --

Query_10003 --

Query_10004 --

Query_10005 --

Query_10001 TIGGNIQTPQLEVSANIEGLEGKLEAPQITGPSFEGDASLKGAKPQGSIRVDASAPQIEGC

Query_10002 --

Query_10003 --

Query_10004 --

Query_10005 --

Query_10001 LSGPSVGVPAPDLDVHGPGGKLNVPKMKVPKFSASGSIGEGAGVDVTLPTGEMTLPGVSGE

Query_10002 ------GSP---

Query_10003 --

Query_10004 --

Query_10005 --

Query_10001 VSLPEISTGGLEGKVKGAKVKTPELIVQKPKISMQDVDLNLGSPKVKGDMKVYAPGVQGDV

Query_10002 ----------------------------------DVDLNVAAPKMKGGVDVTLPKVEGKV

Query_10003 --

Query_10004 --

Query_10005 --

Query_10001 KGPQVAVKGSKVDIEMPNLEG---TWTAPKMKGGFSTPGVKGEGPAVHVALPKGDVSVSGP

Query_10002 KGPGVDIRGPRVDVSGPDAEGHGPEWNLKMPK--FSTPGVKGEGPAVHVALPKGDVSVSGP

Query_10003 --

Query_10004 --

Query_10005 --

Query_10001 RVSVEAPDVNVEGLGGQLKGPDIQLPAVSVKSPKISMPDVDLHAKGPEVKGEWDVTVPKLE

Query_10002 RVSVEAPDVNVEGLGGQLKGPDIQLPAVSVKSPKISMPDVDLHAKGPEVKGEWDVTVPKLE

Query_10003 --

Query_10004 --

Query_10005 --

Query_10001 GELKGPKVDIAAPDVDVHGPDWHLKMPKMKIPKFSVPGFKAEGPEVDVNLPKADVNISAPK

Query_10002 GELKGPKVDIAAPDVDVHGPDWHLKMPKMKIPKFSVPGFKAEGPEVDVNLPKADVNISAPK

Query_10003 --

Query_10004 --

Query_10005 --

Query_10001 VDVHAPDVSIEGPEGKLKGPKFKMPEMNIKAPKISVPDVDLHLKGPHVKGEYDVTTPKVQS

Query_10002 VDVHAPDVSIEGPEGKLKGPKFKMPEMNIKAPKISVPDVDLHLKGPHVKGEYDVTTPKVQS

Query_10003 --

Query_10004 --

Query_10005 --

Query_10001 EIKVPDVELKSARVDIEAPDVDVQGPDWHLKMPKMKTPKFSMPGFKAEGPEVDVNLPKADM

Query_10002 EIKVPDVELKSARVDIEAPDVDVQGPDWHLKMPKMKTPKFSMPGFKAEGPEVDVNLPKADM

Query_10003 --

Query_10004 --

Query_10005 --

Query_10001 DVSGPRVDVEVPDVNVEGPEGKLKGPKFKMPEMNIKAPKISMPDVDLHMKGPKVKGEYDVT

Query_10002 DVSGPRVDVEVPDVNVEGPEGKLKGPKFKMPEMNIKAPKISMPDVDLHMKGPKVKGEYDVT

Query_10003 ---

Query_10004 ---

Query_10005 ---

Query_10001 VPKVEGDLKGPKVDVSAPDVEVHGPDWNLKMPKIKMPKFSMPSLKGEGPELDVNLPKADVD

Query_10002 VPKVEGDLKGPKVDVSAPDVEVHGPDWNLKMPKIKMPKFSMPSLKGEGPELDVNLPKADVD

Query_10003 ---

Query_10004 ---

Query_10005 ---

Query_10001 ISAPKVDLSAPDLSLEGPEGKLKGPKFKMPEMHFKAPKMSLPDVDMDLKGPKMKGSLDVSA

Query_10002 ISAPKVDLSAPDLSLEGPEGKLKGPKFKMPEMHFKAPKMSLPDVDMDLKGPKMKGSLDVSA

Query_10003 ---

Query_10004 ---

Query_10005 ---

Query_10001 PKVEAEMKVPDVDIRGPKVDIKDPHMEGQGPDWSLKMPKMKMPKFTMPSLKAEGPEVDVNL

Query_10002 PKVEAEMKVPDVDIRGPKVDIKDPHMEGQGPDWSLKMPKMKMPKFTMPSLKAEGPEVDVNL

Query_10003 ---

Query_10004 ---

Query_10005 ---

Query_10001 PKADIDIAAPKVDIEAPDVSLEGPEGKLKGPKFKMPEMHFKAPKVSMPDVDLNLKGPKVKG

Query_10002 PKADIDIAAPKVDIEAPDVSLEGPEGKLKGPKFKMPEMHFKAPKVSMPDVDLNLKGPKVKG

Query_10003 ----------------------------------MPDLHLKAPKISMPDVDLNLKGPKVKG

Query_10004 ----------------------------------MPDLHLKAPKISMPDVDLNLKGPKVKG

Query_10005 ---

Query_10001 EMDVSVPKVEGEMKVPDVDIKAPKVGIDVPDVDVQGPDWHLKMPKMKMPKFSMPGFKAEGP

Query_10002 EMDVSVPKVEGEMKVPDVDIKAPKVGIDVPDVDVQGPDWHLKMPKMKMPKFSMPGFKAEGP

Query_10003 DVDVSLPKVEGEIKAPEVDLKGPKVDIDAPDVDVHGPDWHLKMPKIKMPKFSMPGFKAEGP

Query_10004 DVDVSLPKVEGEIKAPEVDLKGPKVDIDAPDVDVHGPDWHLKMPKIKMPKFSMPGFKAEGP

Query_10005 --

Query_10001 EVDVNLPKVDIDVSAPKVDIEAPDVSLEGPEGKLKGPKFKMPEMHFKTPKVSMPDADLNLK

Query_10002 EVDVNLPKADIDIAAPKVDIEAPDVSLEGPEGKLKGPKFKMPEMHFKTPKVSMPDADLNLK

Query_10003 EVDVNLPKADIDVSAPKVDIEGPDVSIEGPEGKLKGPKFKMPEMNIKAPKISMPDVDLNLK

Query_10004 EVDVNLPKADIDVSAPKVDIEGPDVSIEGPEGKLKGPKFKMPEMNIKAPKISMPDVDLNLK

Query_10005 --

Query_10001 GPKLKGDVDVSLPKVEGEIKVPDVGIKGPKVEVGAPDVDVHGPDWHLKMPKMKMPKFSMPG

Query_10002 GPKLKGDVDVSLPKVEGEIKVPDVGIKGPKVEVGAPDVDVHGPDWHLKMPKMKMPKFSMPG

Query_10003 GPKVKGDVDVSLPKVEGEIKAPEVDLKGPKVDIDAPDVDVHGPDWHLKMPKVKMPKFSMPG

Query_10004 GPKVKGDVDVSLPKVEGEIKAPEVDLKGPKVDIDAPDVDVHGPDWHLKMPKVKMPKFSMPG

Query_10005 --

Query_10001 FKAEGPEVDVNLPKADIDVSGPKVDVEVPDVNIEGPDVKVKGPKFKMPEMNIKPQKISMPD

Query_10002 FKAEGPEVDVNLPKADIDVSGPKVDVEVPDVNIEGPDVKVKGPKFKMPEMNIKPQKISMPD

Query_10003 FKAEGPEVDVNLPKADIDVSGPKVDIEGPDVSIEGPEGKLKGPKFKMPEINIKAPKISMPD

Query_10004 FKAEGPEVDVNLPKADIDVSGPKVDIEGPDVSIEGPEGKLKGPKFKMPEINIKAPKISMPD

Query_10005 --

Query_10001 VGLHLKGPKVKGDYDVAVPKVEGEIKAPDVDIKGPKVDVNAPE--VHGPDWHLKMPKM---

Query_10002 VGLHLKGPKVKGDYDVAVPKVEGEIKAPDVDIKGPKVDVNAPE--VHGPDWHLKMPKM---

Query_10003 VDLDLKGPKARGDFDLSVPKTDGVFKSPDVDLKGVRLDLEGPDGKVSGPD--LKMPSLEIS

Query_10004 VDLDLKGPKARGDFDLSVPKTDGVFKSPDVDLKGVRLDLEGPDGKVSGPD--LKMPSLEIS

Query_10005 --

Query_10001 -------------KMPKFSMPGFKA-------EGPEVDVNLPKANIDVSG---------PK

Query_10002 -------------KMPKFSMPGFKA-------EGPEVDVNLPKANIDVSG---------PK

Query_10003 APKLTAPDVDLHLKAPKIGVSGPKLGGGEVDLKGPKVDLEAPSLDVSVGGKGKKSNLKAPD

Query_10004 APKLTAPDVDLHLKAPKIGVSGPKLGGGEVDLKGPKVDLEAPSLDVSVGGKGKKSNLKAPD

Query_10005 ---

Query_10001 VDVDIEAPEGKIKGPKFKM------PSMNIQTHKISMPDVGLNLKAPKLKT-GVDVSLPKV

Query_10002 VDVDIEAPEGKIKGPKFKM------PSMNIQTHKISMPDVGLNLKAPKLKT-GVDVSLPKV

Query_10003 VDVGVTGPDAALKVDVPKMEGEIQAPDLDISSPGIDVEGPDIKVKAPKFKVPGVDVSGPKI

Query_10004 VDVGVTGPDAALKVDVPKMEGEIQAPDLDISSPGIDVEGPDIKVKAPKFKVPGVDVSGPKI

Query_10005 ---

Query_10001 EGELKGPQIDVKAPKMDVDVGDIELEGPDAKLKGPKFKMPEMHFKTPKISMPDVDLNLKGP

Query_10002 EGELKGPQIDVKAPKMDVDVGDIELEGPDAKLKGPKFKMPEMHFKTPKISMPDVDLNLKGP

Query_10003 EGDLKGPSVQA-----NLDAPDINIKGPDAKVKAPSFGI-----SAPHVSMPDVDINLKGP

Query_10004 EGDLKGPSVQA-----NLDAPDINIKGPDAKVKAPSFGI-----SAPHVSMPDVDINLKGP

Query_10005 ---

Query_10001 KVKGDMDVSVPKVEGEMKVPDVDIKAPKVGIDVPDVDVQGPDWHLKMPKMKMPKFSMPGFK

Query_10002 KVKGDMDVSVPKVEGEMKVPDVDIKAPKVGIDVPDVDVQGPDWHLKMPKMKMPKFSMPGFK

Query_10003 KIKGDV--------------------PAVGLEGPDVDLQGPEAKIKFPKFSMPKIGVPGVK

Query_10004 KIKGDV--------------------PAVGLEGPDVDLQGPEAKIKFPKFSMPKIGVPGVK

Query_10005 ---

Query_10001 AEGPEVDVNLPKADIDVSAPKVDIEAPDVSLEGPEGKLKGPKFKMPEMHFKTPKVSMPDVD

Query_10002 AEGPEVDVNLPKADIDVSAPKVDIEAPDVSLEGPEGKLKGPKFKMPEMHFKTPKVSMPDVD

Query_10003 MEGGGAGVHAQLPSLEGG-----LSAPDVKLEGPDVSLKGPGVDLPSVNLSMPKVSGPDLD

Query_10004 MEGGGAGVHAQLPSLEGG-----LSAPDVKLEGPDVSLKGPGVDLPSVNLSMPKVSGPDLD

Query_10005 ---

Query_10001 LNLKGPKVKGSMDVSVPKLEGDLKGPSVDVTMPDVDLECPDAKLK--GPKFKMPEMHFKTP
Query_10002 LNLKGPKVKGSMDVSVPKLEGDLKGPSVDVTMPDVDLECPDAKLK--GPKFKMPEMHFKTP
Query_10003 LNLKGPSLKGDLDASIPGMK---------VHAPGLDLKGVGGKVEMGGDGLKMPGIDVTTA
Query_10004 LNLKGPSLKGDLDASIPGM-----------------------KVEMGGDGLKMPGIDVTTA
Query_10005 ---

Query_10001 -KISMPDVDLNLKGPKVKGEMDVSVPKLEGDLKGPSVDVTMPDVDLECPDAKLKGPKFKMP
Query_10002 -KISMPDVDLNLKGPKVKGEMDVSVPKLEGDLKGPSVDVTMPDVDLECPDAKLKGPKFKMP
Query_10003 LNVGAPDV--TLKGPSLQGDLAVS-----GDIKCPKVSLGAPDLSLEASEGGLKLPQMKLP
Query_10004 LNVGAPDV--TLKGPSLQGDLAVS-----GDIKCPKVSLGAPDLSLEASEGGLKLPQMKLP
Query_10005 ---

Query_10001 EMHFKT-----------------------------PKISMPDVDLNLKGPKVKGSMDVSVP
Query_10002 EMHFKT-----------------------------PKISMPDVDLNLKGPKVKGSMDVSVP
Query_10003 Q---------------------------------RPQISAPDVDFNLEGPKVKGSLGAT--
Query_10004 QFGISTPGSDLDVHIKGPQAAGELQGPGMDVNLRRPQISAPDVDFNLEGPKVKGSLGAT--
Query_10005 ---

Query_10001 KLEGDLKGPSVDVTMP-------DVDL-----ECPDAKLKGPKFK-------------MPE
Query_10002 KLEGDLKGPSVDVTMP-------DVDL-----ECPDAKLKGPKFK-------------MPE
Query_10003 ---GEVKGPTVDFQMPGIKSPGCDVDLPGVNVKLPTGQISGPEIKGDLKGAGVGFHGAAPD
Query_10004 ---GEVKGPTVDFQMPGIKSPGCDVDLPGVNVKLPTGQISGPEIKGDLKGAGVGFHGAAPD
Query_10005 ----------------------------MEKEEETTRELLLPNWQGS---GSHGLTIAQRD

Query_10001 MHFKTPKISM--PDVD--LNLKGPKVKGDMDVSVPKVEGEMKVPDVDIKAPKVGIDV--PD
Query_10002 MHFKTPKISM--PDVD--LNLKGPKVKGDMDVSVPKVEGEMKVPDVDIKAPKVGIDV--PD
Query_10003 ISVKGPSLNMASPEADFGVSLKGPKIKGGVDVS-----GGVSAPDVSLGEGHVSVKG--PG
Query_10004 ISVKGPSLNMASPEADFGVSLKGPKIKGGVDVS-----GGVSAPDVSLGEGHVSVKG--PG
Query_10005 DGVFVQEVLQNSPAARTGVVKEGDQIVGATIYFDNLQSGEVTQLLNTMGHHTVGLKLHRKG

Query_10001 VDVQGP--DWHLKMPKMKMPKFSMPGFKAEGPEVDVNLPKADIDVSAPKVDIEAPDVSLEG

Query_10002 VDVQGP--DWHLKMPKMKMPKFSMPGFKAEGPEVDVNLPKADIDVSAPKVDIEAPDVSLEG

Query_10003 GELKGPQVSSSLNLDASKLA----GGLHFSGPKVEG--VKGQIGLQGPGMSVSGPQGHLDS

Query_10004 GELKGPQVSSSLNLDASKLA----GGLHFSGPKVEG--VKGQIGLQGPGMSVSGPQGHLDS

Query_10005 DRSPEPGQTWTHEVFSSRSSEVVLSTSQPSALECKDQNKPKEATSHAGTISASAPNGLQ--

Query_10001 PEGKLKGPKFKMPEMHFKTPKVSMPDVDFNLKGPKIKGDVDVSAPE---LQGELKGPEMDV

Query_10002 PEGKLKGPKFKMPEMHFKTPKVSMPDVDFNLKGPKIKGDVDVSAPE---LQGELKGPEMDV

Query_10003 GSGKVTFPKMKIPKFAFSGRELVGREVGVDINFPREEASVQASAGEGEWEESEVKLKKSKI

Query_10004 GSGKWH---

Query_10005 ---

Query_10001 KGPKLDVDKP----------EISVEGPEGKWKSPKFKMPDMHFKAPKISMPDIDLHLKSPK

Query_10002 KGPKLDVDKP----------EISVEGPEGKWKSPKFKMPDMHFKAPKISMPDIDLHLKSPK

Query_10003 KMPKFNFSKPKGKGGVTSSPEASISGSKGDLKSSKVSLGSLE--------GEVEAESSSPK

Query_10004 ---

Query_10005 ---

Query_10001 IKGDV-DVDVPKLEADLKSSHVDISGPD-----IDIEGPEGKLKGPKFKMPDMHFKAPNIS

Query_10002 IKGDV-DVDVPKLEADLKSSHVDISGPD-----IDIEGPEGKLKGPKFKMPDMHFKAPNIS

Query_10003 GKFSLFKSKKPRHRSNSFSDEREFSAPSTPTGTLEFEGGEMSLEGGKAKGKHGKLKFGTFG

Query_10004 ---

Query_10005 ---

Query_10001 MPDVDLSLKGPKIKGDVDVSVPEVES-KIKVPDVDI----KGPKVDVKAPDVHGPDWHLKM

Query_10002 MPDVDLSLKGPKIKGDVDVSVPEVES-KIKVPDVDI----KGPKVDVKAPDVHGPDWHLKM

Query_10003 G-------LGSKSKGHYEVTGSDDEAGKLQGSGVSLASSDSGTKVGIQLPEVEVQVSTKKE

Query_10004 ---

Query_10005 ---

```
Query_10001  PKVKMPKFSMPGFKAEGPEVDVNLPKADIDVSAPKVDIEGPDVSIEGPEGKLKGPKFKMPE
Query_10002  PKVKMPKFSMPGFKAEGPEVDVNLPKADIDVSAPKVDIEGPDVSIEGPEGKLKGPKFKMPE
Query_10003  -------------------------------------------------------------
Query_10004  -------------------------------------------------------------
Query_10005  -------------------------------------------------------------

Query_10001  MNIKAPKISMPDVDLNLKGPKVKGDVDVSLPKVEGEIKAPEVDLKGPKVDIDAPDVDVHGP
Query_10002  MNIKAPKISMPDVDLNLKGPKVKGDVDVSLPKVEGEIKAPEVDLKGPKVDIDAPDVDVHGP
Query_10003  -------------------------------------------------------------
Query_10004  -------------------------------------------------------------
Query_10005  -------------------------------------------------------------

Query_10001  DWHLKMPKVKMPKFSMPGFIGRGPRSGCESSQGRH
Query_10002  DWHLKMPKVKMPKFSMPGFIGRGPRSGCESSQGRH
Query_10003  -----------------------------------
Query_10004  -----------------------------------
Query_10005  -----------------------------------
```

STRING Analysis of differentially expressed proteins in old ASCs

sASCs - complete STRING network

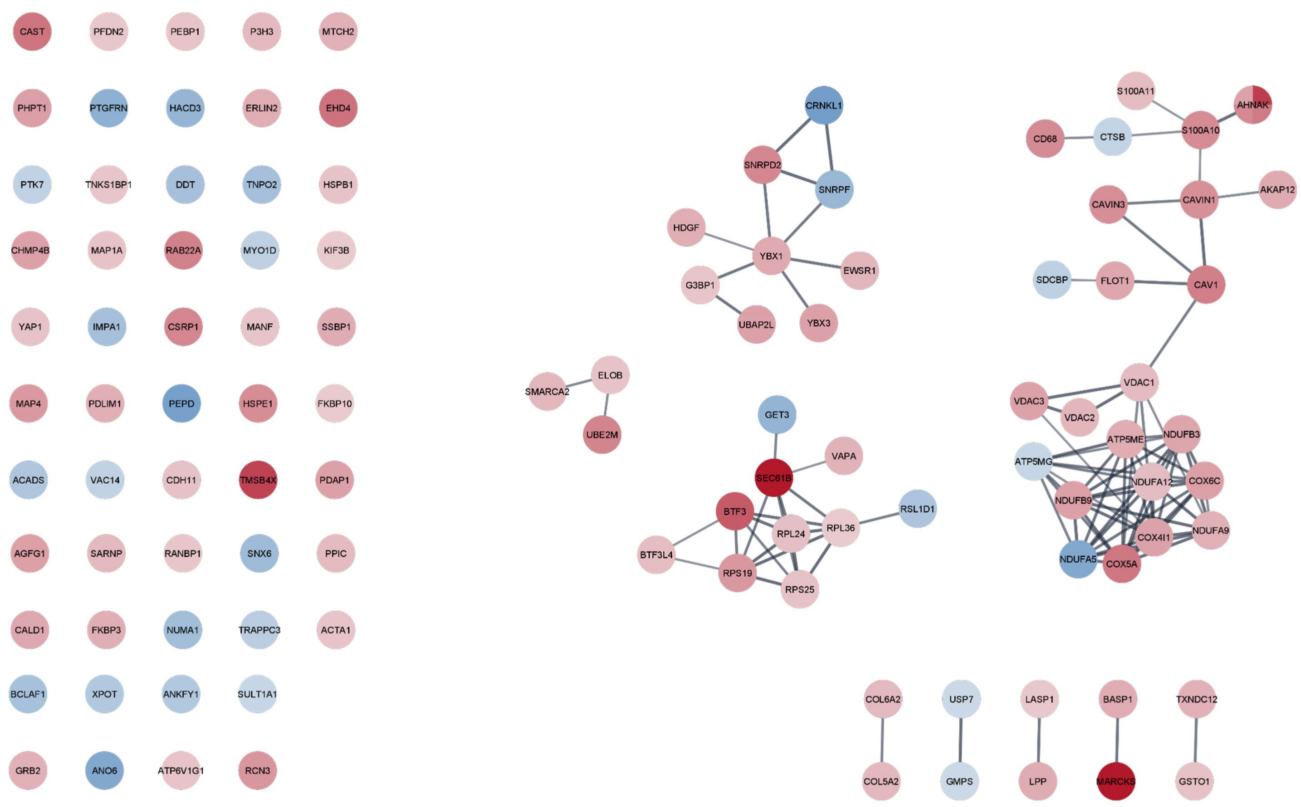

SFig. 1: STRING analysis of differentially expressed proteins in old subcutaneous ASCs
This figure additionally shows the non-clustering proteins as well as small clusters made up by two to three proteins. The differentially expressed proteins (log_2 fold-change minimum ± 0.8, p-value < 0.05) in old s/vASCs were subjected to a STRING analysis using the software Cytoscape (Version 3.9.1). A confidence cut off of 0.7 was employed. The lines between the proteins (nodes) indicate functional and/or physical interactions. The colour of the nodes, red or blue show the up or down regulation of the differentially expressed proteins. Node colour: red upregulation, blue down regulation.

vASCs - complete STRING network

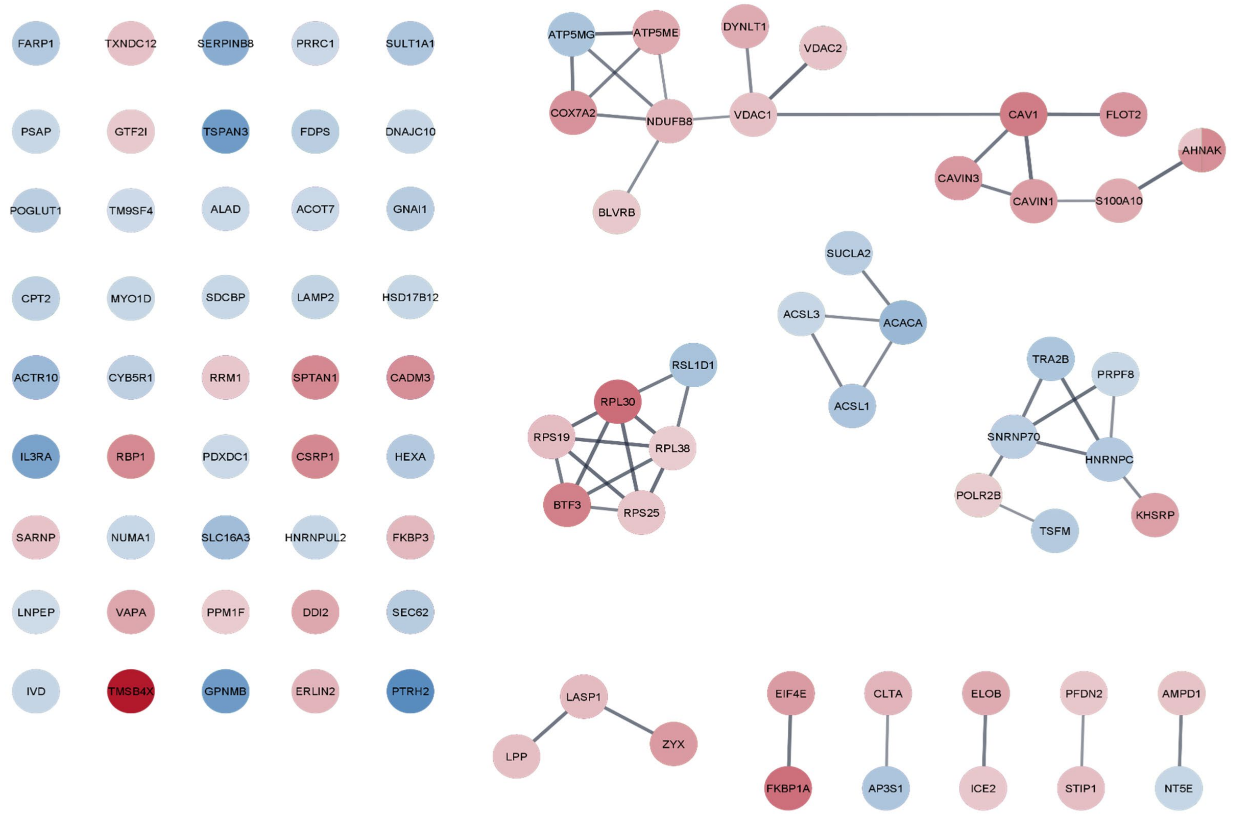

SFig. 2: STRING analysis of differentially expressed proteins in old visceral ASCs
This figure additionally shows the non-clustering proteins as well as small clusters made up by two to three proteins. The differentially expressed proteins (log_2 fold-change minimum ± 0.8, p-value < 0.05) in old s/vASCs were subjected to a STRING analysis using the software Cytoscape (Version 3.9.1). A confidence cut off of 0.7 was employed. The lines between the proteins (nodes) indicate functional and/or physical interactions. The colour of the nodes, red or blue show the up or down regulation of the differentially expressed proteins. Node colour: red upregulation, blue down regulation.

Validation of differential expression of mitochondrial proteins COX5A, NDUFB3, and NDUFB9 by immunoblotting

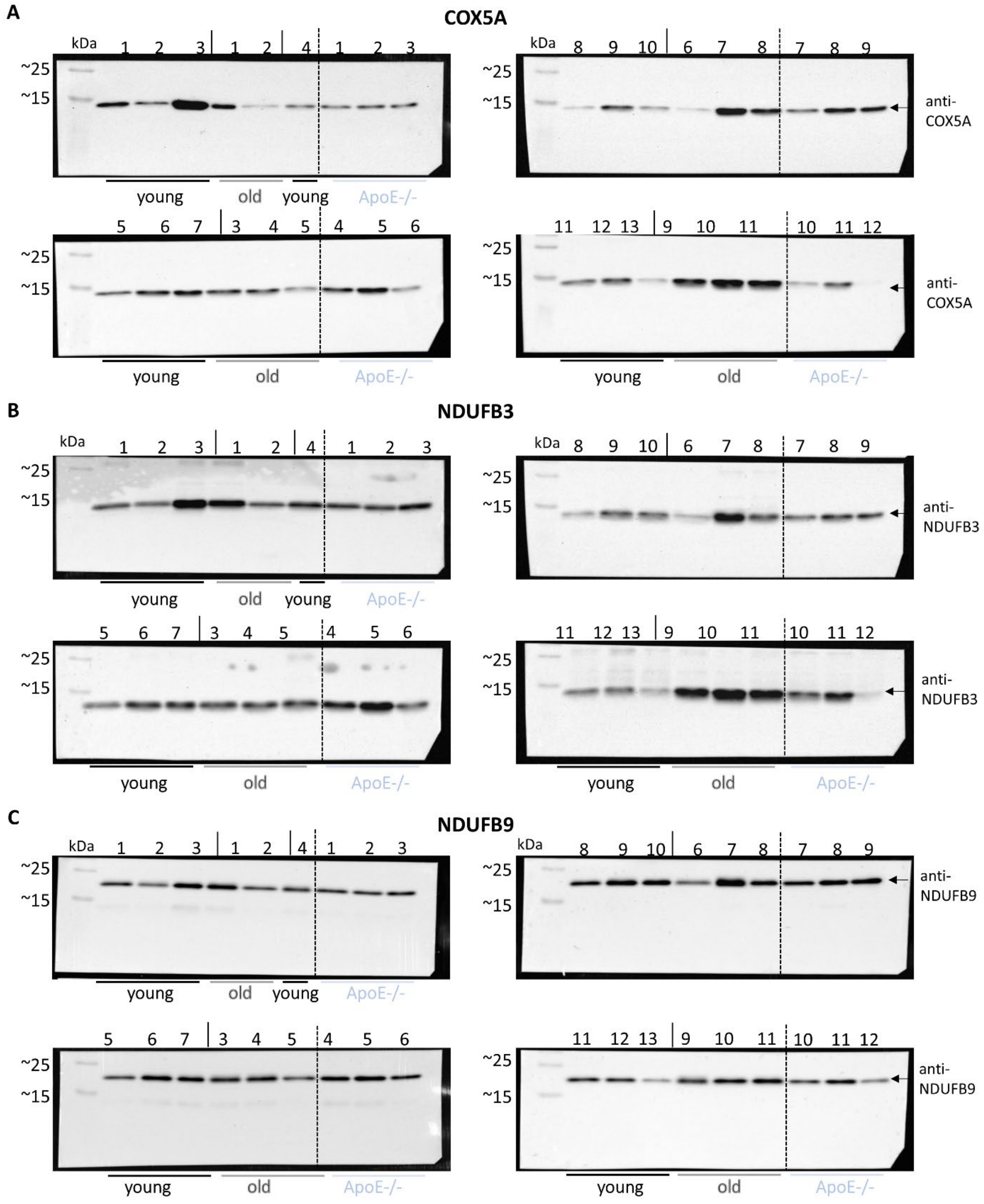

SFig. 3: Western blot membranes using specific antibodies for COX5A, NDUFB3 and NDUFB9

25 µg of total RIPA protein lysates of the samples was used for the western blot analysis. The signal intensity of the corresponding band for each individual protein was normalised to the total protein load of the sample using the Ponceau S staining. The corresponding photos of the Ponceau S stained membranes are shown in SFig 4 and 5. Multiple membranes sections (between ~25 to 10 kDa) were used for antibody hybridisation and analysis. **A)** shows 4 nitrocellulose membrane sections detecting COX5A (anti-COX5A 1:1000). **B)** shows 4 nitrocellulose membrane sections detecting NDUFB3 (anti-NDUFB3 1:1000). **C)** shows 4 nitrocellulose membrane sections detecting NDUFB9 (anti-NDUFB9 1:1000). A Goat-anti-mouse secondary antibody

conjugated to a horse radish peroxidase (Dianova, 1: 10000) was used for all western blot experiments. The arrow indicates the specific band of the target protein. The dotted line marks the ApoE-Knockout sASC lines samples which were not subject to this study. The numbers 1- 11/13/12 correspond to the sample number of the respective sASC groups with n_{young}= 13; n_{old}= 11; $n_{ApoE\text{-}Knockout}$ = 12.

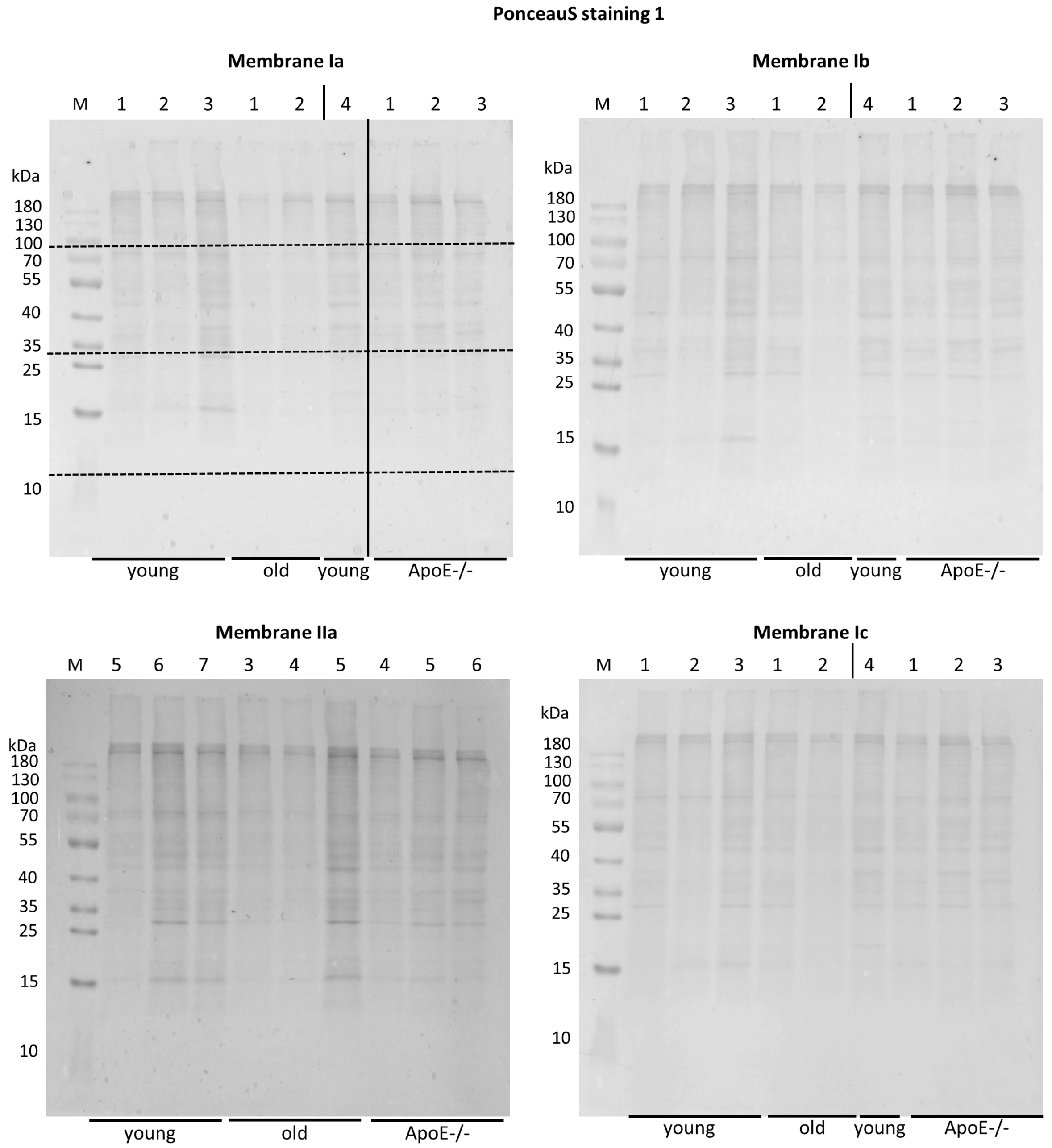

SFig 4: uncut Ponceau S stained nitrocellulose membranes western blots 1

25 µg total protein lysates of the samples were loaded in each lane for the western blot analysis. All membranes were stained with Ponceau S staining and photographed for documentation. Shown as membrane: Ia, b, c; IIa; SFig.5 IIIa, b and Iva, b. For each target protein the following Ponceau S stained membranes were used: COX5A – Ia, IIa, IIIb and IVb; NDUFB3 – Ib, IIa, IIIb and IVb; NDUFB9: Ic, IIa, IIIa and IVa. The dotted lines mark the cutting sites of the nitrocellulose membrane prior to blocking and antibody (hybridisation) incubation. The vertical bold line marks the ApoE-knockout (ApoE-/-) ASC lines which were not considered for this analysis. The numbers 1- 13 correspond to the sample number of the respective sASC group with n_{young}= 13, n_{old}= 11 and $n_{ApoE\text{-/-}}$=12. M indicates the PageRuler™ Prestained Protein Ladder (26616).

PonceauS staining 2

Membrane IIIa

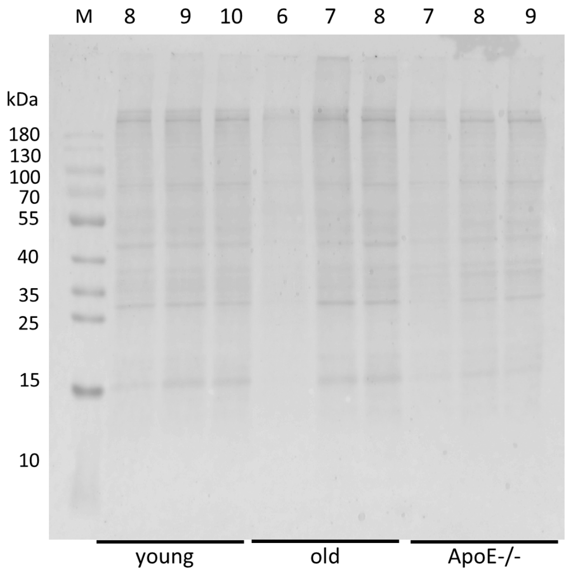

Membrane IIIb

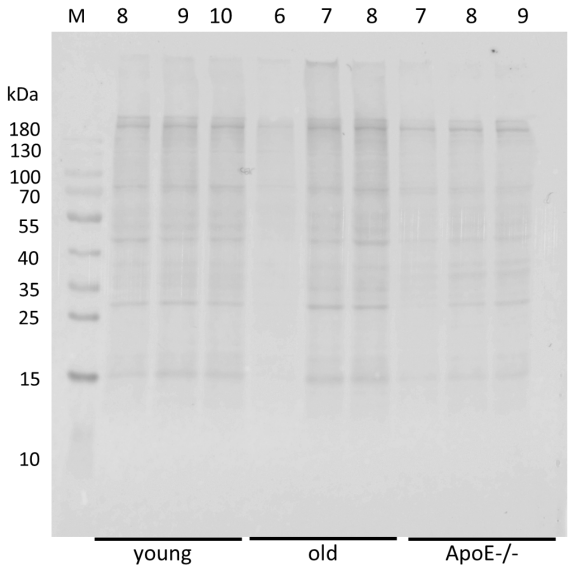

Membrane IVa

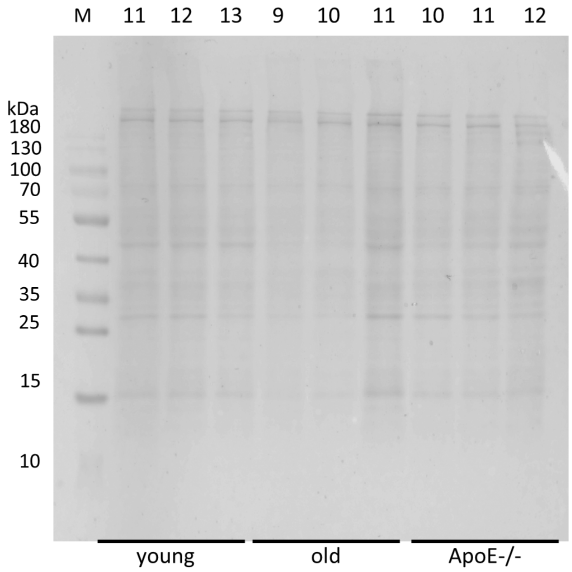

Membrane IVb

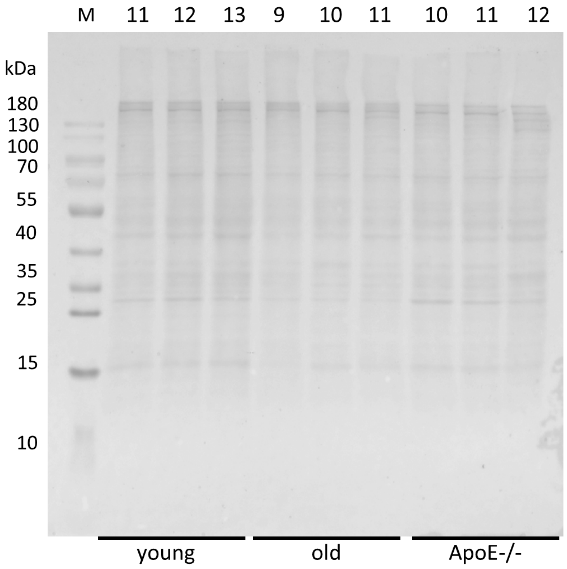

SFig. 5: uncut Ponceau S stained nitrocellulose membranes western blots 2
